# Structure-guided optimization of quinoline inhibitors of *Plasmodium N*-myristoyltransferase†
†The authors declare no competing interests.
‡
‡Electronic supplementary information (ESI) available: Synthesis details and structural characterization of compounds, X-ray data collection and statistics, supplementary Fig. S1–S6. See DOI: 10.1039/c6md00531d


**DOI:** 10.1039/c6md00531d

**Published:** 2016-11-11

**Authors:** Victor Goncalves, James A. Brannigan, Alice Laporte, Andrew S. Bell, Shirley M. Roberts, Anthony J. Wilkinson, Robin J. Leatherbarrow, Edward W. Tate

**Affiliations:** a Department of Chemistry , Imperial College London , London SW7 2AZ , UK . Email: victor.goncalves@u-bourgogne.fr ; Email: e.tate@imperial.ac.uk; b Structural Biology Laboratory , Department of Chemistry , University of York , York YO10 5DD , UK

## Abstract

The parasite *Plasmodium vivax* is the most widely distributed cause of recurring malaria. *N*-Myristoyltransferase (NMT), an enzyme that catalyses the covalent attachment of myristate to the N-terminal glycine of substrate proteins, has been described as a potential target for the treatment of this disease. Herein, we report the synthesis and the structure-guided optimization of a series of quinolines with balanced activity against both *Plasmodium vivax* and *Plasmodium falciparum N*-myristoyltransferase (NMT).

## Abstract 2

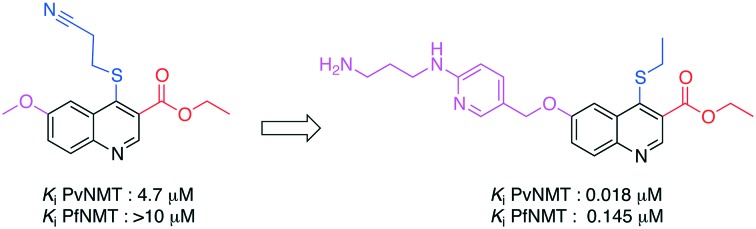
Quinolines with balanced activities against both *Plasmodium vivax* and *Plasmodium falciparum N*-myristoyltransferase were identified.

## Introduction

*N*-Myristoyltransferase (NMT, EC 2.3.1.97) catalyses the transfer of the fatty acid chain C_14:0_ from myristoyl coenzyme A (MyrCoA) to the N-terminal glycine residue of substrate proteins.^1^ This co- and post-translational modification is ubiquitous in eukaryotic organisms and plays a central role in a variety of cellular processes such as the addressing and reversible anchoring of proteins to membranes.^2^,^3^ The *N*-terminal myristate chain can also participate in the stabilization of the tertiary structure of proteins and form part of recognition elements that establish protein–protein interactions.^4^,^5^ Due to the essential functions of myristoylation, the modulation of NMT activity has emerged as an attractive strategy in the treatment of various pathologies. In particular, NMT has been validated as a pharmacological target in fungal infections,^6^ in a range of parasitic diseases caused by *Trypanosoma*,^7^*Leishmania*^8^ and *Plasmodium*^9^–^11^ protozoa and filarial nematodes,^12^ and as a potential target for the treatment of cancer.^13^

The first reported NMT inhibitors were obtained through rational design strategies, by mimicking the structure of peptide substrates,^14^,^15^ or by designing non-hydrolysable MyrCoA analogues.^16^,^17^

Later, novel families of NMT inhibitors were identified by high throughput screening (HTS) efforts.^7^,^18^–^20^ A selection of different chemotypes are under active development against NMT for numerous indications (Fig. S1‡), and an NMT inhibitor has recently entered the Medicines for Malaria Venture (MMV) development pipeline for the treatment of the lethal form of malaria caused by *Plasmodium falciparum*. We reported in 2012 the discovery of a *Plasmodium vivax* NMT (PvNMT) inhibitor based on a quinoline scaffold.^21^*P. vivax* is the most frequent parasite responsible for the recurring form of malaria, whose transmission and infection occur through the bite of *Anopheles* mosquitos.^22^ Vivax malaria strongly impacts the quality of life of infected patients who experience cyclical fever and weakness episodes and represents a severe burden in endemic countries, due to cost of treatment and loss of productivity.^23^

**Fig. 1:**
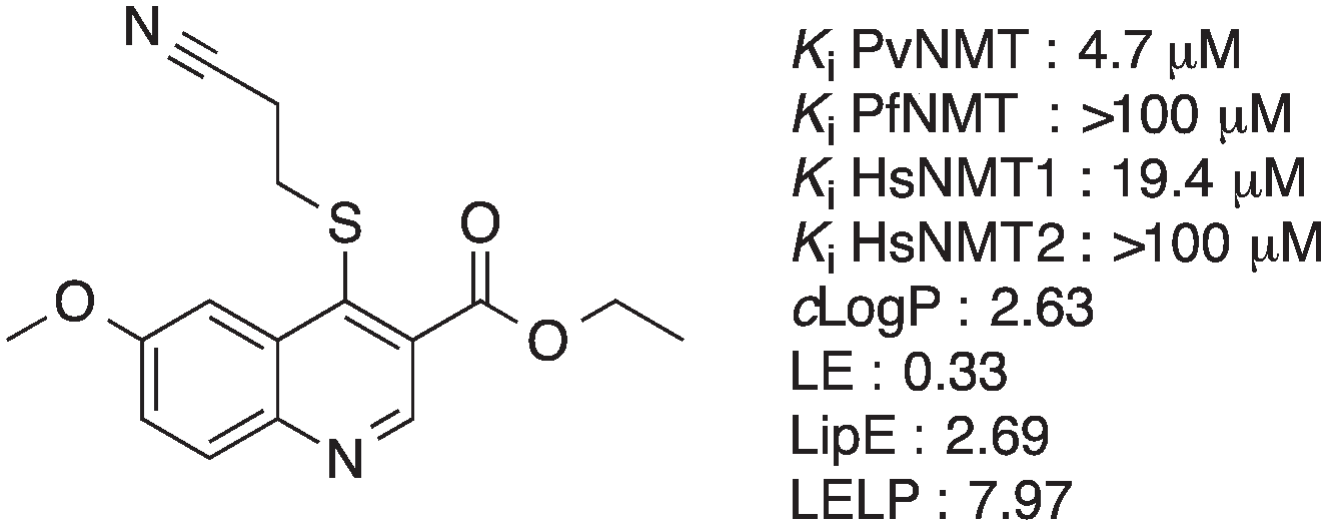
Structure of the optimized molecule **1**. clog *P* was determined with ChemAxon, which can be obtained from ; http://www.chemaxon.com/. LE: ligand efficiency, LE = [–log(*K*_i_)](1.374)/(no. of heavy atoms).^24^ LipE: lipophilic efficiency, LipE = –log(*K*_i_) – clog *P*.^24^ LELP : ligand efficiency dependent lipophilicity, LELP = clog *P*/LE.^25^ Here, we report how our attempts to improve the potency of this series of compounds through a structure-guided strategy led to the identification of nanomolar inhibitors of *N*-myristoyltransferase, active against both PvNMT and PfNMT.

Our initial work on the quinoline series resulted in the elucidation of the crystal structure of a hit compound in complex with PvNMT and the identification of an optimized molecule, ethyl 4-((2-cyanoethyl)thio)-6-methoxyquinoline-3-carboxylate **1**, displaying micromolar inhibitory potency against PvNMT, some selectivity *versus* the human NMT isoforms and reasonable physico-chemical properties (Fig. 1). Unfortunately, these compounds showed no activity against the *N*-myristoyltransferase of *Plasmodium falciparum*, the species causing the most virulent and deadly form of the disease.

## Results and discussion

### Crystal structure of lead compound **1** in complex with *Plasmodium vivax* NMT

**Fig. 2:**
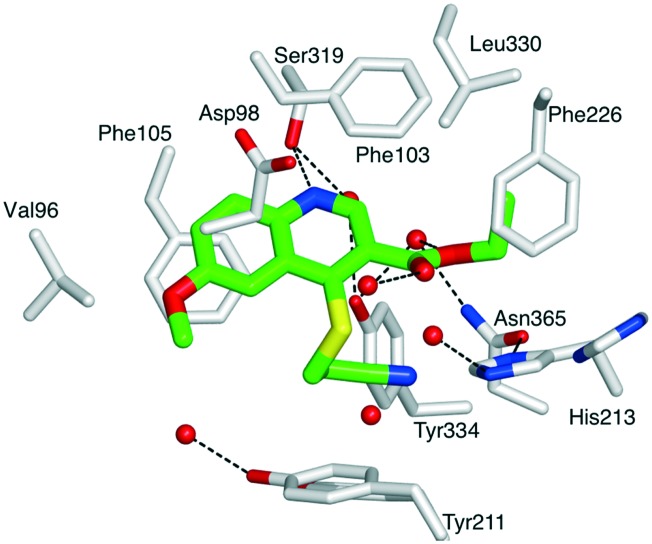
Structure of quinoline **1** in a ternary complex with *P. vivax* NMT and *S*-(2-oxo)pentadecylCoA (NHM) (PDB accession code: ; 5G1Z). View of **1** in chain A of PvNMT in cylinder format and colored by atom: carbon (green), oxygen (red), nitrogen (blue), sulphur (yellow). The side chains of residues located within 4 Å of **1** are displayed as cylinders and labelled. Water molecules are shown as red spheres. Polar interactions with PvNMT are represented as dashed lines. Y211 and H213 are shown in two alternate conformations. For a stereoview, see Fig. S2.‡

First, compound **1** was co-crystallized with PvNMT and *S*-(2-oxo)pentadecyl-coenzyme A, a non-hydrolysable myristoyl-coenzyme A analogue (Fig. 2),^26^ and the structure of the resulting ternary complex was solved by X-ray crystallography. Overall, **1** adopts a binding mode similar to the original hit compound, 3-butyl-4-((2-cyanoethyl)thio)-6-methoxy-2-methylquinoline (MRT00057965), which we identified in 2012 by high throughput screening.^21^ The quinoline fits into a hydrophobic pocket, where it establishes parallel-displaced π–π stacking interactions with the adjacent side-chains of Phe103 and Phe105. The binding of the quinoline ring is reinforced by the establishment of an H-bond between its nitrogen and the hydroxyl function of Ser319. Additionally, the nitrile function of the 2-cyanoethylthioether chain appears to interact with the imidazole ring of His213 (distance ∼3.0 Å). The ligand is modelled bound to the protein at half maximal occupancy due to the low affinity of binding. This is mirrored in the two orientations of residues His213 and Tyr211, corresponding to the bound and free forms (Fig. 2). Interestingly, the ester function, introduced to improve the solubility and log *P* of the hit compound, also establishes polar interactions with PvNMT, through water-mediated H-bonds.

Based on the above structure, the optimization of the 3-, 4- and 6-positions of the quinoline ring was undertaken.

### Optimization of the substituent in position 4 of the quinoline ring

It is well known that 2-cyanoethylthioethers can be cleaved in the presence of weak bases such as ammonia through a retro-Michael reaction.^27^ Although **1** was fully stable in the phosphate buffered solutions used for enzymatic assay, some degradation was observed during its chemical synthesis, suggesting it may represent a hurdle in the future development of this series of compounds. Additionally, structural data indicate that the H-bond formed between the nitrile and His213 may not be critical for the binding of the inhibitor. This prompted us to investigate whether the 2-cyanoethylthioether could be advantageously replaced by a more stable substituent in the 4-position of the quinoline ring.

**Scheme 1:**
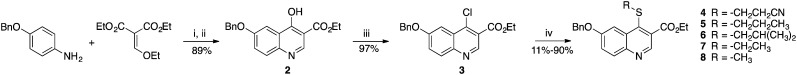
Synthetic pathway to compounds **2–8**. Reagents and conditions: (i) 140 °C, 1 h; (ii) Ph_2_O, 240 °C, 2 h; (iii) POCl_3_, 110 °C, 20 min; (iv) R-SH or R-SNa, K_2_CO_3_, THF or DMF, RT, 1.5–24 h.

First, the linear alkyl isostere **5**, with an *n*-propyl chain in place of the 2-cyanoethyl, was synthesized. In this series of molecules, the original 6-methoxy group present in the optimized hit **1** was replaced by a benzyloxy. Indeed, the 6-benzyloxy derivative **4**, initially prepared as a synthetic intermediate to explore the role of the 6-position, was found to provide an 8-fold improvement in activity compared to the original methoxy compound, probably due to the establishment of edge-to-face π–π interactions with the side chains of residues Phe105 and Tyr211 (Table 1). The intermediate ethyl 6-(benzyloxy)-4-hydroxyquinoline-3-carboxylate **2** was obtained through the thermal condensation and cyclization of 6-benzyloxyaniline and ethyl ethoxymethylenemalonate, and subsequently transformed into its 4-chloro derivative **3** by treatment with POCl_3_ (Scheme 1).^28^ Finally, nucleophilic aromatic substitution with thiols or sodium thiolates provided the 4-thioalkyl derivatives **4–8**.

**Table 1:** Structure and biochemical activity of compounds **4–19** on PvNMT, PfNMT and HsNMTs

Entry	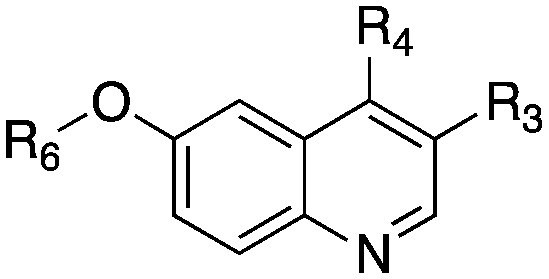			*K*_i_^app^ (μM)^*a*^				clog *P*^*b*^	LipE^*c*^
	R_3_	R_4_	R_6_	PvNMT	PfNMT	HsNMT1	HsNMT2		
**1**	COOEt	SCH_2_CH_2_CN	Me	4.74 ± 0.24 (ref. 21)	>100	19.43 ± 0.86 (ref. 21)	>100 (ref. 21)	2.63	2.69
**4**	COOEt	SCH_2_CH_2_CN	Bn	0.55 ± 0.06	>10	>10	n.d.	4.36	1.90
**5**	COOEt	SCH_2_CH_2_CH_3_	Bn	2.08 ± 0.14	1.10 ± 0.08	3.36 ± 0.37	>10	5.46	0.22
**6**	COOEt	SCH(CH_3_)_2_	Bn	2.51 ± 0.16	1.99 ± 0.30	4.84 ± 0.64	>10	5.25	0.35
**7**	COOEt	SCH_2_CH_3_	Bn	0.44 ± 0.05	0.67 ± 0.04	1.56 ± 0.22	4.23 ± 0.53	4.94	1.42
**8**	COOEt	SCH_3_	Bn	4.14 ± 0.41	5.90 ± 0.60	n.d.	n.d.	4.69	0.69
**9**	COOEt	SOCH_2_CH_3_	Bn	1.75 ± 0.12	5.65 ± 1.24	3.60 ± 0.30	>10	3.30	2.46
**10**	COOEt	SO_2_CH_2_CH_3_	Bn	1.78 ± 0.47	>10	>10	>10	3.41	2.34
**11**	CH_2_OH	SCH_2_CH_3_	Bn	4.19 ± 0.55	>10	>10	>10	3.81	1.57
**12**	CH_2_OCH_2_CH_3_	SCH_2_CH_3_	Bn	2.92 ± 0.15	2.69 ± 0.51	>10	>10	4.81	0.72
**13**	CO–NHCH_2_CH_3_	SCH_2_CH_3_	Bn	0.34 ± 0.05	0.96 ± 0.11	1.05 ± 0.115	>10	4.01	2.46
**14**	CO–N(CH_2_CH_3_)_2_	SCH_2_CH_3_	Bn	0.41 ± 0.06	0.94 ± 0.09	1.02 ± 0.13	3.53 ± 0.40	4.59	1.80
**15**	CO-*N*-pyrrolidine	SCH_2_CH_3_	Bn	0.96 ± 0.13	1.52 ± 0.29	1.36 ± 0.21	>10	4.28	1.74
**16** ^*d*^	CO–NH–Ph	SCH_2_CH_3_	Bn	n.d.	n.d.	n.d.	n.d.	5.67	
**17** ^*d*^	CO–NH-2-thiazole	SCH_2_CH_3_	Bn	n.d.	n.d.	n.d.	n.d.	5.01	
**18**	CO–NHCH_2_CH_2_OH	SCH_2_CH_3_	Bn	2.84 ± 0.38	8.00 ± 3.98	>10	>10	3.19	2.36
**19**	CO*-N*-morpholine	SCH_2_CH_3_	Bn	0.20 ± 0.02	0.33 ± 0.02	0.10 ± 0.01	0.59 ± 0.05	3.66	3.03

^*a*^Apparent *K*_i_ values of tested compounds on the enzymatic activity of recombinant *P. vivax* NMT, *P. falciparum* NMT and *H. sapiens* NMT isoforms 1 and 2. Each *K*_i_ is the mean ± SD from duplicates. n.d. : not determined.

^*b*^clog *P* were determined with ChemAxon.

^*c*^LipE: lipophilic efficiency, LipE = –log(*K*_i_) – clog *P*.

^*d*^No biological data available because of the limited solubility of the compounds in assay buffer.

The inhibitory potency of **4–8** was assessed using a fluorogenic enzymatic assay for PvNMT activity.^21^ Compounds were also tested on PfNMT, the enzyme present in *P. falciparum*,^29^ and on the human isoforms HsNMT1 and HsNMT2.^30^ Replacing the 2-cyanoethyl thioether with an *n*-propyl thioether (compound **5**) had a limited impact on PvNMT activity, confirming that the nitrile function is not required for high affinity binding to the enzyme. Interestingly, this modification was also associated with a >10-fold improvement in activity *versus* PfNMT. Indeed, while the original hit compound and compound **4** were totally inactive on PfNMT up to 100 μM, **5** displayed a *K*_i_ of 1.1 μM, similar to the value measured with PvNMT. These differences in terms of binding affinity between PvNMT and PfNMT, two enzymes that share 80% identity, are difficult to rationalize. The only residue in the inhibitor-binding pocket that differs between these NMTs is Tyr334 (Pv numbering), which is replaced by a phenylalanine in PfNMT. Based on X-ray data, this residue does not establish polar interactions with the nitrile function of the inhibitor. Therefore, how such a minor modification causes significant changes in binding affinity remains to be defined. Unfortunately, despite substantial crystallisation efforts, no X-ray structure of PfNMT alone, or in complex with inhibitors, has been solved so far.

Subsequent modification of the length and nature of the thioether chain allowed us to identify the ethylthioether derivative **7**, as the compound having the best potency against both PvNMT and PfNMT (Table 1).

**Scheme 2:**
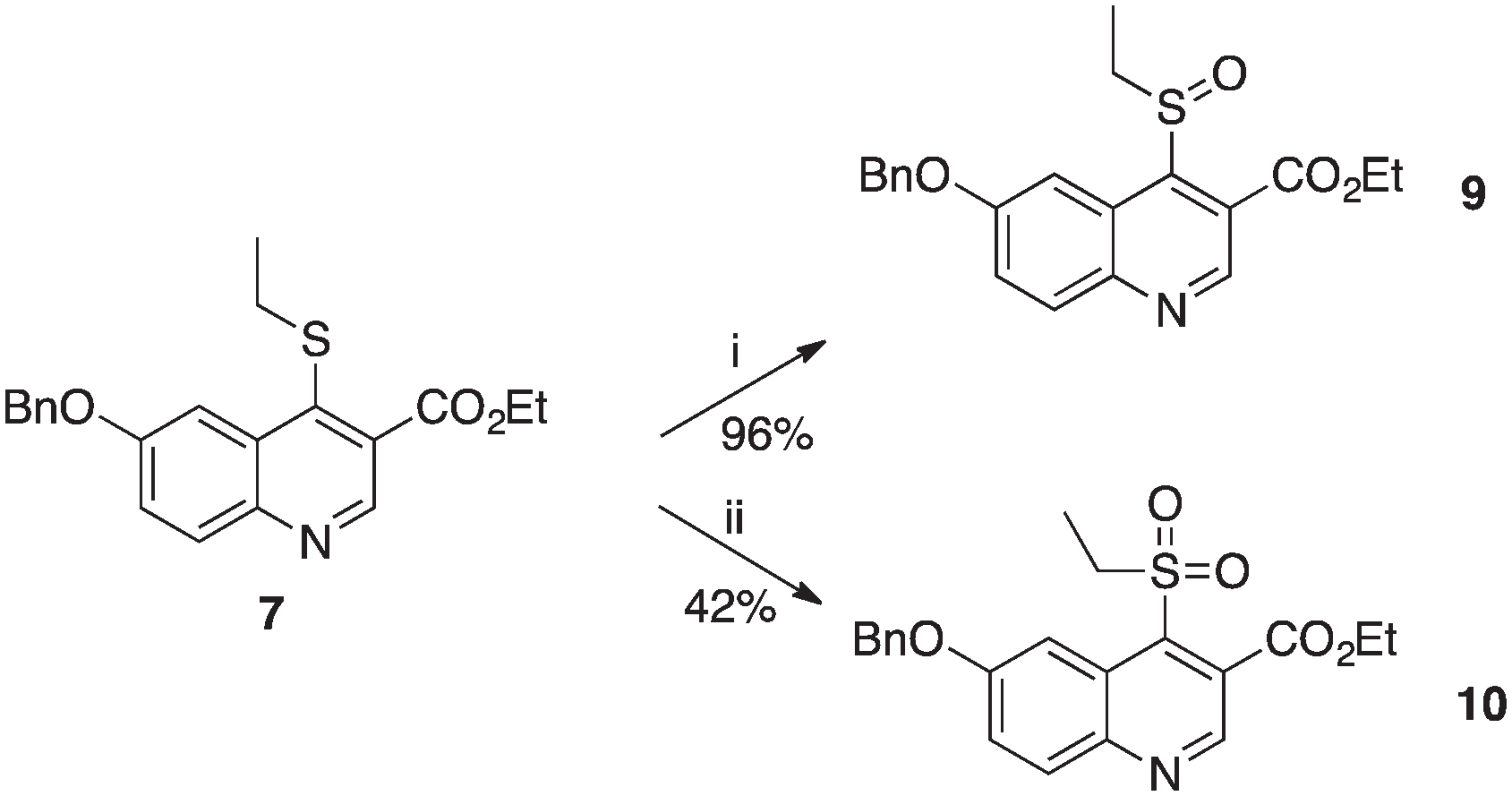
Syntheses of sulfoxide and sulfone derivatives **9** and **10**. Reagents and conditions: (i) *m*-CPBA, DCM, –78 °C, 4 h; (ii) *m*-CPBA, DCM, 0 °C, 2.5 h.

The X-ray structure of the complex between **1** and PvNMT shows that a cluster of water molecules surrounds the sulfur atom of the thioether chain. We attempted to establish polar contacts with these water molecules through the synthesis of the sulfoxide and sulfone derivatives **9** and **10** respectively, which were obtained by mCPBA-mediated oxidation of **7** at –78 °C and 0 °C respectively (Scheme 2). Unfortunately, both appeared to be less active than the original thioether **7**. At this stage, it is difficult to determine whether this reduced activity is due to unfavourable steric clashes/displacement of water molecules or if it originates from the electron-withdrawing effects of the sulfone and sulfoxide on the quinoline ring, which may reduce the strength of the H-bond between the quinoline nitrogen and Ser319 of PvNMT.

It is worth noting that, although **9** and **10** were less active than the corresponding alkyl analogue, their LipE values (based on PvNMT data) were significantly higher due to their enhanced hydrophilicity. However, the ethyl thioether was selected as the preferred substituent for the rest of the study because of its better synthetic tractability.

### Replacement of the ethyl ester group by amides

**Scheme 3:**
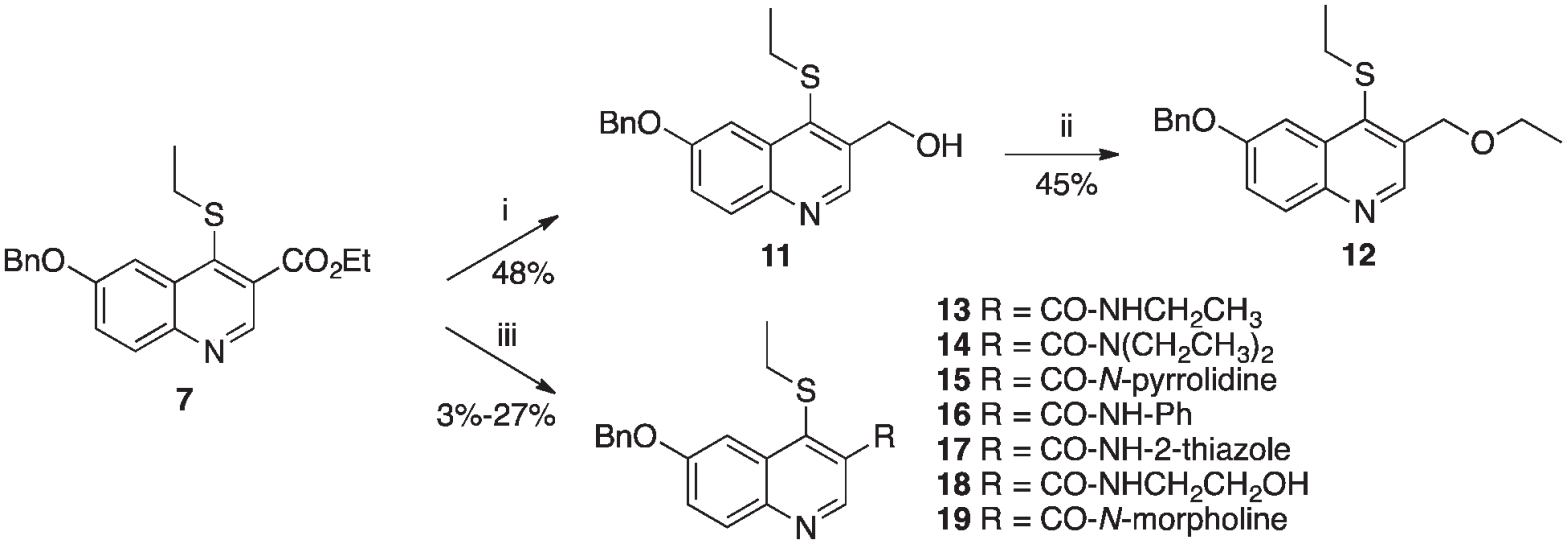
Syntheses of alcohol, ether and amide derivatives **11–19**. Reagents and conditions: (i) LiAlH_4_, THF, 0 °C, 1 h; (ii) *t*BuOK, DMSO, RT then EtI, 60 °C, 1 h; (iii) AlMe_3_, amine, toluene, –15 °C then **7**, RT, 24 h.

The original hit compound, MRT00057965, possessed an *n*-butyl chain in position 3 of the quinoline ring. In our previous study,^21^ this group was replaced by an ethyl ester in order to reduce the lipophilicity of the compound. While this modification succeeded in improving the hydrophilicity of the quinoline without significantly impacting the affinity of the molecule for PvNMT, it represents a potential liability with respect to hydrolysis *in vivo*. In an attempt to identify more suitable substituents, the ester **7** was reduced to form the alcohol **11**, which was subsequently alkylated to provide the ethyl ether derivative **12** (Scheme 3). Both compounds showed a 6- to 10-fold reduction in inhibitory activity. Subsequently, the ester was replaced by an amide. In order to allow direct comparison with the ester series, the ethyl amide **13** was synthesized initially, from compound **7** according to Weinreb's procedure (Scheme 3).^31^ Encouragingly, **13** showed the same inhibitory profile as **7**, with a *K*_i_ of 0.34 μM and 0.96 μM on PvNMT and PfNMT respectively, and a 3-fold and 30-fold selectivity *versus* HsNMT1 and HsNMT2 respectively.

Superposition of the three quinoline–PvNMT complexes present in the asymmetric unit of the crystal shows that the ethyl ester group is flexible and able to adopt different conformations in a relatively large pocket, suggesting that the ethyl chain is suboptimal and that the enzyme may tolerate larger substituents. This hypothesis was confirmed through the synthesis of the *N*,*N*-diethylamide **14** and the *N*-pyrrolidine amide derivative **15** which showed similar levels of potency as the *N*-ethylamide **13** across the different enzymes. Substitution of the carboxamide with small aromatic rings (phenyl, **16**, or 2-thiazole **17**), which could interact with nearby residues Phe226 and His213, led to the formation of poorly soluble compounds that could not be evaluated. Finally, in order to decrease the lipophilicity of the series, and potentially establish novel electrostatic interactions with the enzyme, amides substituted with polar functions were synthesized. While the 2-hydroxyethylamide derivative **18** displayed a significant loss of affinity, the *N*-morpholine derivative **19** showed an improved potency, as well as a significantly reduced clog *P*.

**Fig. 3:**
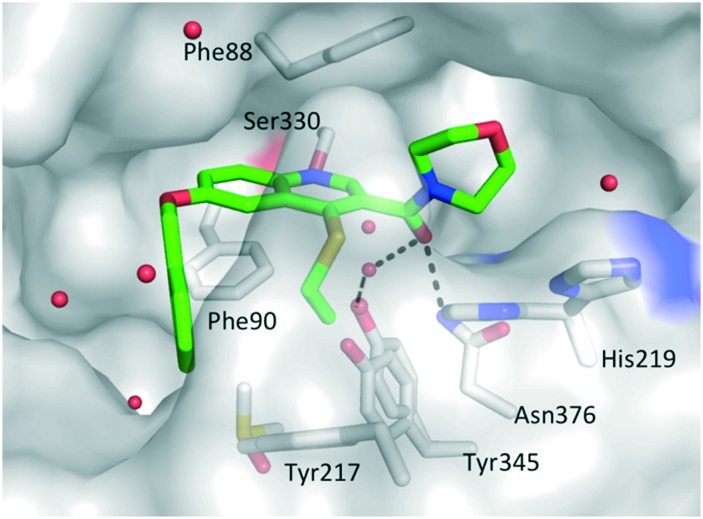
Structure of quinoline **19** in a ternary complex with LmNMT and MyrCoA (PDB accession code: ; 5G20). **19** is shown in cylinder format and colored by atom: carbon (green), oxygen (red), nitrogen (blue) and sulphur (yellow). A transparent LmNMT surface is shown in grey. The side chains of selected LmNMT residues located within 4 Å of **19** are displayed as grey sticks, and labeled. Water molecules are shown as red spheres. Polar interactions with LmNMT and solvent are represented as black dashed lines. For a stereo view, see Fig. S2.‡

A ternary crystal structure of **19** in complex with *Leishmania major* NMT (LmNMT) and NHM was obtained (Fig. 3).^7^,^32^ LmNMT and PvNMT share 39% sequence identity and have been used interchangeably for structural biology studies, with LmNMT offering the more robust platform for rapid crystallography. As before, the ethyl thioether chain in position 4 seems to stack against the phenol group of Tyr217 (Tyr211 in PvNMT) and the position of histidine side chain continues to adopt two orientations. The main difference with compound **1** arises from the (*N*-morpholine)carboxamide group. The orientation of the amide undergoes a 160° twist compared to the initial orientation of the ester, allowing the carbonyl to H-bond with Asn376 and *via* a water molecule with Tyr345 (Fig. 3). This change is associated with the full rotation of Phe232 (Phe226 in PvNMT) to accommodate the presence of the morpholine ring (Fig. S3‡). It remains to be determined if these changes are specific to compound **19** or if they occur with all amide derivatives.

### Investigation of the substituent in position 6 of the quinoline ring

**Fig. 4:**
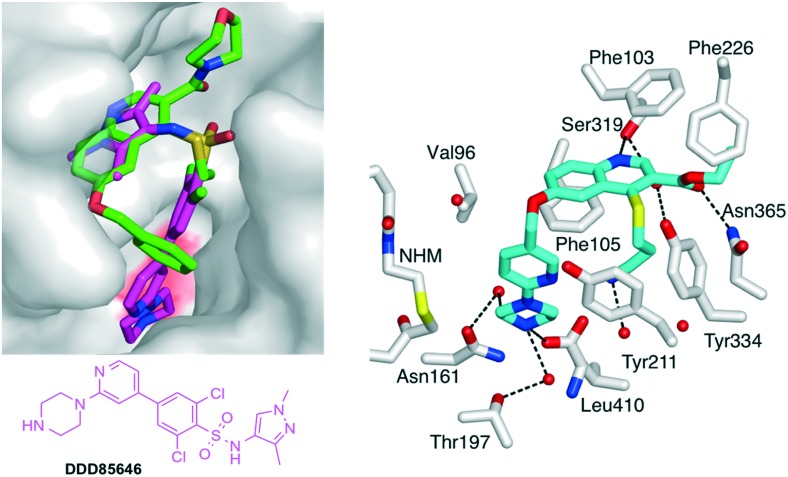
(Left) View of **19** (green carbons) in LmNMT in cylinder format. The surface of LmNMT is shown in grey. **19** is superimposed with **DDD85646** (2,6-dichloro-4-(2-piperazin-1-ylpyridin-4-yl)-*N*-(1,3,5-trimethyl-1*H*-pyrazol-4-yl)-benzenesulfonamide; magenta carbons), a known NMT inhibitor. The surface above the C-terminal leucine of NMT is colored in red. (Right) Crystal structure of **26** (cyan carbons) in complex with PvNMT and non-hydrolizable MyrCoA (pdb accession code: ; 5G22). Selected side chains of PvNMT are shown in cylinder (format) and labeled according to PvNMT numbering. Dashed lines indicate electrostatic/H-bond interactions. For further views of **26** in complex with PvNMT and LmNMT, see Fig. S4, S5 & S6.‡

Finally, the role of the substituent located on position 6 of the quinoline ring was explored. The benzyloxy-present in all the above compounds sits at the top of a narrow cavity, which leads to the catalytic C-terminal leucine of *N*-myristoyltransferase (Fig. 4). Virtually all of the known families of NMT inhibitors interact with the C-terminal residue, which is a leucine in fungi, Plasmodium and Leishmania but is a valine in *T. brucei* and a glutamine in human NMTs. This interaction is usually formed by an amino group in the inhibitor that establishes a strong ionic bond with the C-terminal NMT carboxylate.^7^,^9^,^18^,^33^ However, inhibitors containing imidazoles^34^ or neutral functions^35^ have also been reported to establish stabilizing interactions with the C-terminus.

With the objective of further increasing the inhibitory potency of the quinoline series, novel molecules were designed, in which the 6-benzyloxy moiety was replaced by longer substituents elaborating an amine group at their extremity. By analogy with **DDD85846**, the benzyloxy was first replaced by a (6-(piperazin-1-yl)pyridin-3-yl)methoxy group (Fig. 4).

**Scheme 4:**
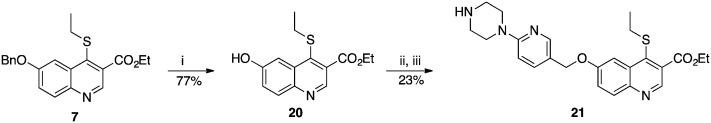
Synthesis of compound **21**. Reagents and conditions: (i) methanesulfonic acid 10% (v/v) in TFA, 60 °C, 4 h; (ii) (6-(4-Boc-piperazin-1-yl)pyridin-3-yl)methanol, DEAD, PPh_3_, THF, RT, 16 h; (iii) TFA 20% in DCM, RT, 40 min.

Compound **21** was prepared from **7** in three steps: 6-hydroxyquinoline intermediate **20** was first formed under acidic conditions, and was subsequently condensed with (6-(4-Boc-piperazin-1-yl)pyridin-3-yl)methanol by a Mitsunobu reaction (Scheme 4). Deprotection of the piperazine afforded **21**, with an overall yield after purification of 18%. A similar strategy was employed to synthesize the derivatives **22–25**, in which the piperazine was replaced with alternative cyclic or linear diamines (Table 2). These modifications were introduced to identify the most suitable linker and to take into account potential differences between LmNMT and the intended target, PvNMT.

**Table 2:** Structure and biochemical activity of compounds **21–26** on PvNMT, PfNMT and HsNMTs

Entry	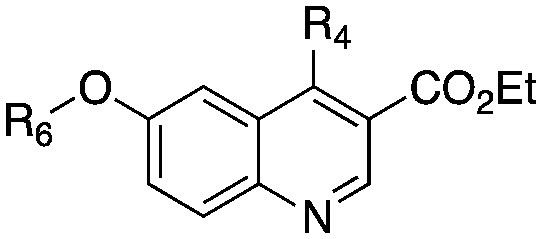		*K*_i_^app^ (μM)^*a*^				clog *P*^*b*^	LipE^*c*^
	R_4_	R_6_	PvNMT	PfNMT	HsNMT1	HsNMT2		
**7**	SCH_2_CH_3_	Bn-	0.44 ± 0.05	0.67 ± 0.04	1.56 ± 0.22	4.23 ± 0.53	4.94	1.42
**21**	SCH_2_CH_3_	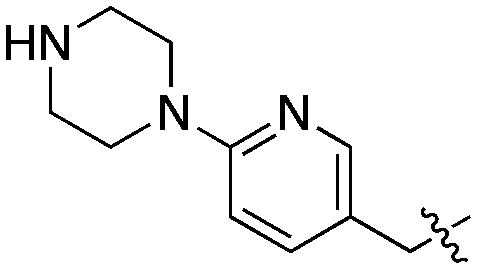	0.04 ± 0.01	0.24 ± 0.02	0.04 ± 0.01	0.10 ± 0.01	3.89	3.53
**22**	SCH_2_CH_3_	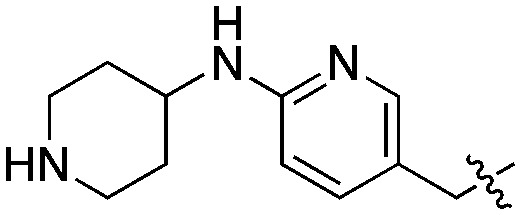	0.09 ± 0.01	0.40 ± 0.05	0.08 ± 0.01	0.69 ± 0.08	3.43	3.64
**23**	SCH_2_CH_3_	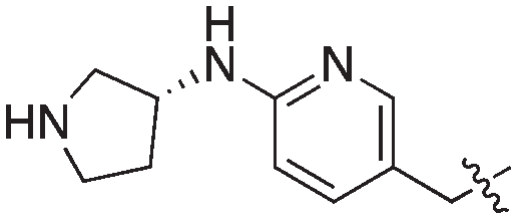	0.14 ± 0.01	0.96 ± 0.10	0.05 ± 0.01	0.37 ± 0.06	3.37	3.49
**24**	SCH_2_CH_3_	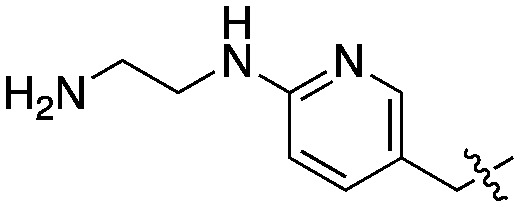	0.28 ± 0.02	1.25 ± 0.08	0.05 ± 0.01	0.41 ± 0.07	2.99	3.57
**25**	SCH_2_CH_3_	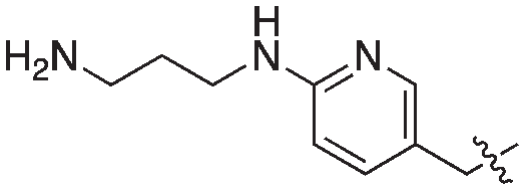	0.02 ± 0.01	0.15 ± 0.02	0.008 ± 0.002	0.10 ± 0.01	3.05	4.69
**26**	SCH_2_CH_2_CN	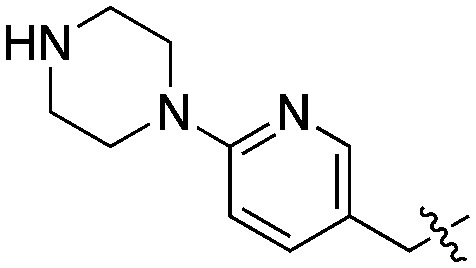	0.10 ± 0.01	3.31 ± 1.15	0.37 ± 0.03	1.35 ± 0.20	3.30	3.72

^*a*^Apparent *K*_i_ values of tested compounds on the enzymatic activity of recombinant *P. vivax* NMT, *P. falciparum* NMT and *H. sapiens* NMT isoforms 1 and 2. Each *K*_i_ is the mean ± SD from duplicates.

^*b*^clog *P* was determined with ChemAxon.

^*c*^LipE: lipophilic efficiency.

Compared to the initial benzyloxy **7**, the 4-piperazine-pyridin-3-yl derivative **21** showed a dramatic improvement in potency against PvNMT (>10-fold, *K*_i_ = 38 nM), and a similar, while more limited, effect on PfNMT (*K*_i_ = 242 nM). This increase in potency was also associated with a loss of selectivity *versus* HsNMT1 and HsNMT2. Replacing the piperazine with a 4-amino-piperazine, a pyrrolidine, or ethylenediamine did not improve activity against PvNMT or PfNMT. The most promising results were obtained with the 1,3-propanediamine derivative **25**, which inhibited PvNMT with a *K*_i_ of 18 nM.

Crystal structures were determined of **26**, in complex with first PvNMT to explore the basis of potency, and then with LmNMT, to allow comparison of its binding mode with that of **19** (Fig. 4, S4, S5 & S6‡). As expected, an ionic interaction is observed between the piperazine and the C-terminal leucine. This complex is further stabilized by direct and water-mediated H-bonds of the piperazine with Thr197 and Asn161. The pyridine ring also establishes a stabilizing polar interaction with the phenol of Tyr211. The structure of **26** bound to LmNMT (PDB code ; 5G21) is closely superimposable but shows a slightly different orientation of Tyr211, possibly due to multiple orientations of the nitrile tail (Fig. S4‡). It is worth noting that the quinoline plane of compound **26** adopts a slightly tilted orientation compared to the one occupied by **19** in LmNMT (Fig. S5 & S6‡). We assume that this potentially unfavourable change is related to an ionic interaction with LmNMT, which constrains the molecule to reduce the distance between the piperazine nitrogen and Leu421 carboxylate. This may explain the higher potency of the more flexible 1,3-propanediamine derivative **25**.

## Conclusion

Starting from the crystal structure of compound **1** in PvNMT, the positions 3-, 4- and 6-substituents of the quinoline ring were sequentially modified, with the aim of improving chemical stability, solubility and affinity of this series of compounds for *N*-myristoyltransferases. Interestingly, while the primary objective of this work was to increase the inhibitory potency of the quinoline series for PvNMT, we observed that the substitution of the 2-cyanoethythioether moiety by an alkyl thioether resulted in a significant improvement in the activity towards *Plasmodium falciparum* NMT, affording novel lead compounds with balanced activities against both PvNMT and PfNMT. Moreover, controlling the lipophilicity as part of the optimization process allowed us to identify novels inhibitors with significantly improved lipophilic efficiency.

The low selectivity over host NMT may limit the potential to progress this series to lead optimisation.^36^ However, we recently reported that ligands that induce conformational changes in Tyr211 of PvNMT were selective for *Plasmodium* NMTs over human NMT,^9^ and further investigation of the 4-position of the quinoline, which is in contact with Tyr211, may be a fruitful area for future exploration.

Finally, we expect that the crystal structures obtained as part of this work, along with information on the quinoline binding mode, will support the development of even more potent *N*-myristoyltransferase inhibitors, based on a quinoline core alone or hybridized with other series of inhibitors targeting NMT.^18^,^35^

## Notes

The authors declare no competing financial interest.

## Supplementary Material

Supplementary information
