# Supplementary material for: Structure-guided optimization of quinoline inhibitors of Plasmodium N-myristoyltransferase
Source: Medchemcomm. 2016 Nov 11;8(1):191–7. doi: 10.1039/c6md00531d (PMC5463734; doi:10.1039/c6md00531d)
Supplement: Supplementary file 1 [file MD-008-C6MD00531D-s001.pdf]

Electronic Supplementary Material (ESI) for MedChemComm.
This journal is © The Royal Society of Chemistry 2016

SUPPORTING INFORMATION

Structure-guided optimization of quinoline inhibitors of *Plasmodium N*-myristoyltransferase†

Victor Goncalves, ‡*[a] James A. Brannigan, [b] Alice Laporte, [a] Andrew S. Bell, [a] Shirley M. Roberts, [b] Anthony J. Wilkinson,[b] Robin J. Leatherbarrow ††[a] and Edward W. Tate*[a]

[a] *Department of Chemistry, Imperial College London, London SW7 2AZ, United Kingdom*

[b] *Structural Biology Laboratory, Department of Chemistry, University of York, York YO10 5DD, United Kingdom*

*E-mail: victor.goncalves@u-bourgogne.fr

*E-mail: e.tate@imperial.ac.uk

†Electronic Supplementary Information (ESI) available: Synthesis details and structural characterization of compounds, X-ray data collection and statistics. See DOI: 10.1039/x0xx00000x

‡ Current address: ICMUB, Université Bourgogne Franche-Comté, Dijon, 21000, France.

†† Current address: Liverpool John Moores University, Egerton Court, 2 Rodney Street, Liverpool, L1 2UA, UK.

CHEMICAL SYNTHESIS.

General methods

All chemicals were purchased from Sigma-Aldrich Ltd (Gillingham, UK), Acros Organics (Geel, Belgium) and Alfa Aesar (Heysham, UK) and used without further purification. Moisture sensitive reactions were performed under nitrogen or argon atmosphere using dried glassware. Silica gel column flash chromatography was performed on an Isolera (Biotage, Uk) automated apparatus with ZIP or SNAP silica cartridges (Biotage, UK). NMR spectra were recorded on 400 MHz Bruker instruments at room temperature and were referenced to TMS or residual solvent signals. Data are presented as follows: chemical shift, multiplicity (br s = broad singlet, s = singlet, d = doublet, t = triplet, q = quartet, m = multiplet) and integration.
The purity of title compounds was verified by RP-HPLC on a Waters 2767 system equipped with a photodiode array and an ESI mass spectrometer. Separation was achieved using a XBridge C_{18} (5 µm, 4.6 mm × 100 mm) column, equipped with an XBridge C18 guard column (5 µm, 4.6 mm × 20 mm). The following elution method was used: Gradient of H_2O and MeOH (solvents were supplemented with 0.1% formic acid): 0-10 min 5-98% MeOH, 10-12 min 98% MeOH, 12-13 min 98 to 5% MeOH, 13-17 min 5% MeOH. Flow rate: 1.2 mL/min. Purity of tested compounds was ≥ 95%, unless specified. Mass spectra and accurate mass data were obtained at the Chemistry Department Mass Spectrometry Service (Imperial College London) by electrospray ionization (ESI) or electron impact ionization (EI).

Synthetic procedures

Ethyl 6-(benzyloxy)-4-hydroxyquinoline-3-carboxylate 2. 4-(benzyloxy)aniline (14.0 g, 0.07 mol) and diethyl (ethoxymethylene)malonate (14.1 mL, 0.07 mol) were stirred for 15 min at 90 °C, and for 45 min at 130 °C. The brown mixture was cooled to room temperature and triturated in cyclohexane (200 mL). The precipitate was isolated by filtration on Buchner, washed with cyclohexane and dried under vacuum to provide diethyl 2-(((4-(benzyloxy)phenyl)amino)methylene)malonate as a beige powder (24.5 g, 86%). This intermediate (8 g, 21.6 mmol) was added over 5 min to a solution of diphenylether (20 mL) at 240 °C in an open flask. The suspension was stirred for 2 h at 240 °C and cooled to 40-50 °C. *n*-hexane (20 mL) was added and the mixture was stirred for 10 min. The brown precipitate was filtered on Buchner, washed with *n*-hexane (3 × 30 mL), DCM (2 × 10 mL) and dried over vacuum to provide **2** as a beige powder (6.2 g, 89%). ^1H NMR (400 MHz, DMSO) δ 8.48 (s, 1H), 7.31-7.70 (m, 9H), 5.20 (s, 2H), 4.29 (q, J = 7.2, 3H),1.27 (t, J = 7.2, 3H).

Ethyl 6-(benzyloxy)-4-chloroquinoline-3-carboxylate 3. Ethyl 6-(benzyloxy)-4-hydroxyquinoline-3-carboxylate **2** (2.81 g, 8.70 mmol) and $POCl_3$ (3.5 mL, 37 mmol) were heated to reflux for 1 h. The solution was cooled to room temperature and poured slowly into a stirred solution of ice and saturated ammonium hydroxide (0-5 °C). Extraction of the solution with ethyl acetate provided a brown oil, which was purified by flash chromatography on silica (45 g silica; 100% *n*-hexane to 65/35 *n*-hexane/ethyl acetate). **3** was isolated as a yellow solid (2.93 g, 97%). ^1H NMR (400 MHz, $CDCl_3$) δ 9.04 (s, 1H), 8.01 (d, J = 9.2, 1H), 7.65 (d, J = 2.6, 1H), 7.58 – 7.29 (m, 6H), 5.19 (d, J = 6.1, 2H), 4.48 (q, J = 7.1, 2H), 1.45 (t, J = 7.1, 3H).

Ethyl 6-(benzyloxy)-4-((2-cyanoethyl)thio)quinoline-3-carboxylate 4. 3-mercaptopropanenitrile was prepared according to litterature [1] and kept at -20 °C under argon. Ethyl 6-(benzyloxy)-4-chloroquinoline-3-carboxylate **3** (327 mg, 0.95 mmol), 3-mercaptopropanenitrile (0.184 mL, 1.9 mmol) and K_2CO_3 (263 mg, 1.9 mmol) were dissolved in dry THF (3 mL) and stirred at room temperature for 1.5 h under argon. The solution was extracted with EtOAc, washed with brine, dried over $MgSO_4$ and evaporated under reduced pressure. The residue was purified by

flash chromatography on silica (gradient of *n*-hexane/EtOAc from 95/5 to 60/40) to give **4** as a white powder (298 mg, 80%). ^{1}H NMR (400 MHz, $CDCl_3$) δ 8.91 (s, 1H), 8.09 (d, *J* = 9.2, 1H), 7.94 (d, *J* = 2.8, 1H), 7.57 (dd, *J* = 9.2, 2.8, 1H), 7.51 (d, *J* = 7.2, 2H), 7.47 – 7.29 (m, 3H), 5.34 (s, 2H), 4.49 (q, *J* = 7.1, 2H), 3.02 (t, *J* = 7.1, 2H), 2.37 (t, *J* = 7.1, 2H), 1.45 (t, *J* = 7.1, 3H). ^{13}C NMR (101 MHz, $CDCl_3$) δ 166.52, 158.42, 146.10, 145.13, 138.70, 136.25, 131.98, 130.56, 128.83, 128.32, 127.39, 124.85, 117.90, 105.53, 70.48, 62.35, 31.94, 18.68, 14.29. HRMS (ESI) *m*/*z* calcd for $C_{22}H_{20}N_2O_3S$ $[M + H]^+$ 393.1273, found 393.1272.

Ethyl 6-(benzyloxy)-4-(propylthio)quinoline-3-carboxylate 5. 5 was prepared from **3** (50 mg, 0.15 mmol), propane thiol (0.07 mL, 0.60 mmol) and K_2CO_3 (42 mg, 0.30 mmol) in DMF (2 mL). The residue was purified by flash chromatography on silica (95/5 to 70/30 *n*-hexane/EtOAc) to give **5** as a yellow oil (46.2 mg, 0.12 mmol, 80%). ^{1}H NMR (400 MHz, $CDCl_3$) δ 8.86 (s, 1H), 8.09 (d, *J* = 9.2, 1H), 7.95 (d, *J* = 2.8, 1H), 7.57 – 7.30 (m, 6H), 5.28 (s, 2H), 4.48 (q, *J* = 7.1, 2H), 2.78 (t, *J* = 7.3, 2H), 1.55 – 1.39 (m, 5H + H_2O), 0.91 (t, *J* = 7.3, 3H). ^{13}C NMR (101 MHz, $CDCl_3$) δ 158.00, 145.65, 136.22, 130.48, 128.76, 128.26, 127.50, 124.37, 105.99, 70.45, 62.03, 38.85, 23.35, 14.27, 13.29. HRMS (EI) *m*/*z* calcd for $C_{22}H_{23}NO_3S$ $[M\ ]^+$ 381.1399, found 381.1393.

Ethyl 6-(benzyloxy)-4-(isopropylthio)quinoline-3-carboxylate 6. 6 was prepared using the previous method from **3** (50 mg, 0.15 mmol), 2-propanethiol (27 µL, 0.30 mmol) and K_2CO_3 (42 mg, 0.30 mmol) in DMF (2 mL). **6** was isolated after flash chromatography as a yellow oil (35.7 mg, 62.5%). ^{1}H NMR (400 MHz, $CDCl_3$) δ 8.86 (s, 1H), 8.07 (d, *J* = 9.1, 1H), 7.96 (d, *J* = 2.8, 1H), 7.60 – 7.30 (m, 6H), 5.28 (s, 2H), 4.48 (q, *J = 7.2,* 2H), 3.32 (hept, *J* = 6.7, 1H), 1.44 (t, *J* = 7.2, 3H), 1.14 (d, *J* = 6.7, 6H). ^{13}C NMR (101 MHz, $CDCl_3$) δ 166.98, 157.91, 145.85, 144.59, 141.62, 136.33, 131.89, 131.49, 131.04, 128.75, 128.34, 128.19, 127.78, 127.43, 124.14, 106.29, 70.39, 61.95, 41.12, 23.48, 14.28. HRMS (EI) *m*/*z* calcd for $C_{22}H_{23}NO_3S$ $[M]^+$ 381.1399, found 381.1392.

Ethyl 6-(benzyloxy)-4-(ethylthio)quinoline-3-carboxylate 7. 7 was prepared using the previous method from **3** (300 mg, 0.88 mmol), ethanethiol (0.13 mL, 3.52 mmol) and K_2CO_3 (250 mg, 1.76 mmol) in DMF (3 mL). **7** was isolated after flash chromatography as a yellow oil (290 mg, 90%). ^{1}H NMR (400 MHz, $CDCl_3$) δ 8.86 (s, 1H), 8.05 (d, *J* = 9.2, 2H), 7.94 (d, *J* = 2.8, 1H), 7.61 – 7.31 (m, 6H), 5.27 (s, 2H), 4.48 (q, *J* = 7.2, 2H), 2.83 (q, *J* = 7.4, 2H), 1.44 (t, *J* = 7.2, 3H), 1.13 (t, *J* = 7.4, 3H). ^{13}C NMR (101 MHz, $CDCl_3$) δ 157.96, 145.87, 136.25, 131.44, 130.52, 128.75, 128.24, 127.53, 124.24, 106.00, 70.45, 61.99, 30.93, 15.03, 14.27. HRMS (EI) *m*/*z* calcd for $C_{21}H_{21}NO_3S$ $[M]^+$ 367.1242, found 367.1240.

Ethyl 6-(benzyloxy)-4-(methylthio)quinoline-3-carboxylate 8. 8 was prepared using the previous method from **3** (70 mg, 0.20 mmol), sodium methanethiolate (20 mg, 0.60 mmol) and K_2CO_3 (27.6 mg, 0.20 mmol) in DMF (2 mL). The reaction mixture was stirred at room temperature for 24 h. **8** was isolated after flash chromatography (100/0 to 70/30 *n*-hexane/EtOAc) as a yellow oil (8 mg, 11%). ^{1}H NMR (400 MHz, $CDCl_3$) δ 8.87 (s, 1H), 8.06 (d, *J* = 9.2, 1H), 7.94 (d, *J* = 2.8, 1H), 7.55 – 7.30 (m, 6H), 5.27 (s, 2H), 4.49 (q, *J* = 7.2, 2H), 2.39 (s, 3H), 1.45 (t, *J* = 7.2, 3H), ^{13}C NMR (101 MHz, $CDCl_3$) δ 166.74, 158.00, 146.11, 136.19, 131.47, 129.80, 129.49, 128.75, 128.27, 127.58, 124.28, 105.64, 70.49, 62.02, 29.70, 19.56, 14.25. MS (ESI) *m*/*z* calcd for $C_{20}H_{19}NO_3S$ $[M+H]^+$354.43, found 354.19.

Ethyl 6-(benzyloxy)-4-(ethylsulfinyl)quinoline-3-carboxylate 9. A solution of **7** (100 mg, 0.27 mmol) in DCM (3 mL) was cooled to -78°C under an argon atmosphere. *m*CPBA (75.6 mg, 0.33 mmol, 77% purity) was added portionwise and the mixture was stirred for 4 h at -78 °C. The mixture was extracted with DCM, washed with a solution of saturated sodium bicarbonate and brine, dried over $MgSO_4$ and concentrated under reduced pressure. The residue was purified by flash chromatography (gradient of hexane/EtOAc from 90/10 to 60/40) to give **9** as a viscous yellow oil (99 mg, 96%). ^{1}H NMR (400 MHz, $CDCl_3$) δ 9.05 (s, 1H), 8.81 (s, 1H), 8.10 (d, *J* = 9.3, 1H), 7.58 (dd, *J*

= 2.8, 9.3, 1H), 7.49 (d, *J* = 7.2, 2H), 7.43 – 7.29 (m, 3H), 5.26 (dd, 2H), 4.45 (q, *J* = 7.1, 2H), 3.40 – 3.27 (m, 1H), 3.24 – 3.12 (m, 1H), 1.51 – 1.40 (m, 6H). ^{13}C NMR (101 MHz, $CDCl_3$) δ 164.96, 157.23, 149.42, 146.11, 146.01, 136.17, 131.73, 128.63, 128.17, 127.67, 126.09, 125.65, 122.99, 103.76, 70.40, 62.55, 48.65, 14.24, 8.81. HRMS (EI) *m*/*z* calcd for $C_{21}H_{21}NO_4S$ $[M]^+$ 383.1191, found 383.1187.

Ethyl 6-(benzyloxy)-4-(ethylsulfonyl)quinoline-3-carboxylate 10. A solution of **7** (67 mg, 0.18 mmol) in DCM (3 mL) was cooled to 0°C under an argon atmosphere. *m*CPBA (84.1.6 mg, 0.36 mmol, 77% purity) was added portionwise and the mixture was stirred for 2.5 h at 0°C. The mixture was extracted with DCM, washed with a solution of saturated sodium bicarbonate and brine, dried over $MgSO_4$ and concentrated under reduced pressure. The residue was purified by flash chromatography (gradient of hexane/EtOAc from 95/5 to 65/35) to give **10** as a yellow oil (30.3 mg, 42%). ^{1}H NMR (400 MHz, $CDCl_3$) δ 8.80 (s, 1H), 8.23 (d, *J* = 2.7, 2H), 8.13 (d, *J* = 9.3, 1H), 7.59 (dd, *J* = 9.3, 2.7, 1H), 7.49 (d, *J* = 7.2, 2H), 7.44 – 7.29 (m, 3H), 5.31 (s, 2H), 4.48 (q, *J* = 7.2, 2H), 3.19 (q, *J* = 7.5, 2H), 1.42 (t, *J* = 7.2, 3H), 1.27 (t, *J* = 7.5, 3H). ^{13}C NMR (101 MHz, $CDCl_3$) δ 170.45, 168.30, 159.21, 143.00, 136.07, 134.99, 129.37, 128.89, 128.73, 127.76, 123.82, 121.51, 104.34, 103.34, 71.07, 64.09, 45.80, 14.09, 9.64. HRMS (EI) *m*/*z* calcd for $C_{21}H_{21}NO_5S$ $[M]^+$ 399.1140, found 399.1140.

[6-(benzyloxy)-4-(ethylsulfanyl)quinolin-3-yl]methanol 11. $LiAlH_4$ (102 mg, 2.7 mmol) was added portionwise to a stirred solution of **7** (100 mg, 0.27 mmol) in THF (3 mL) at 0 °C, under argon. The suspension was stirred for 1 h at 0 °C. The reaction was quenched by the addition of ice and NaOH 10% (v/v) and extracted with ethyl acetate. The organic layer was washed with brine, dried over $MgSO_4$, and evaporated under reduced pressure. The crude product was purified by flash chromatography (gradient from 50/50 hexane/EtOAc to 100% EtOAc). **11** was isolated as a yellow powder (44 mg, 48%). ^{1}H NMR (400 MHz, $CDCl_3$) δ 8.85 (s, 1H), 8.07 (d, *J* = 9.1, 1H), 7.84 (d, *J* = 2.7, 1H), 7.64 – 7.30 (m, 6H), 5.27 (s, 2H), 5.08 (s, 2H), 2.73 (q, *J* = 7.4, 2H), 1.11 (t, *J* = 7.4, 3H). ^{13}C NMR (101 MHz, $CDCl_3$) δ 128.78, 128.26, 127.43, 105.53, 70.48, 62.20, 30.60, 15.27. HRMS (EI) *m*/*z* calcd for $C_{19}H_{19}NO_2S$ $[M]^+$ 325.1137, found 325.1135.

6-(benzyloxy)-3-(ethoxymethyl)-4-(ethylsulfanyl)quinoline 12. To a solution of **11** (50 mg, 0.15 mmol) in DMSO (1 mL) was added *t*BuOK (17 mg, 0.15 mmol). The solution was stirred for 20 min at room temperature. Iodoethane (13 µL, 0.17 mmol) was added and the mixture was stirred at 60 °C for 1 h. The solution was cooled to room temperature and extracted with ethyl acetate. The organic phase was washed with brine, dried over $MgSO_4$ and concentrated. Purification by flash chromatography (gradient from 95/5 to 70/30 *n*-hexane/EtOAc) afforded **12** as a yellow oil (23.8 mg, 45%). ^{1}H NMR (400 MHz, $CDCl_3$) δ 8.89 (s, 1H), 8.20 (s, 1H), 7.88 (d, *J* = 2.7, 1H), 7.56 – 7.29 (m, 6H), 5.28 (s, 2H), 4.93 (s, 2H), 3.65 (q, *J* = 7.0, 2H), 2.73 (q, *J* = 7.4, 2H), 1.29 (t, *J* = 7.0, 4H), 1.11 (t, *J* = 7.4, 3H). ^{13}C NMR (101 MHz, $CDCl_3$) δ 157.59, 147.98, 143.82, 140.57, 136.53, 135.97, 131.41, 130.96, 129.74, 128.71, 128.12, 127.47, 122.52, 105.75, 70.34, 69.61, 66.31, 30.48, 15.27, 15.21. HRMS (EI) *m*/*z* calcd for $C_{21}H_{23}NO_2S$ $[M]^+$ 353.1450, found 353.1461.

6-(benzyloxy)-N-ethyl-4-(ethylsulfanyl)quinoline-3-carboxamide 13. To a solution of $AlMe_3$ (2 M in hexanes, 80 µL, 0.16 mmol) in dry toluene (1 mL), at -15 °C, was added ethylamine (2 M in THF, 76 µL, 0.15 mmol). The resulting solution was stirred for 20 min at -15 °C and 45 min at room temperature. **7** (50 mg, 0.14 mmol) was added and the mixture was stirred at 110°C for 24 h. Water (2 mL) and sodium hydroxide 10% (m/v) (0.5 mL) were added to the solution and the product was extracted with ethyl acetate. The organic phase was washed with brine, dried and concentrated under reduced pressure. The residue was purified by flash chromatography (gradient from 50/50 *n*-hexane/EtOAc to 100% EtOAc) to give **13** as a white powder (10.5 mg, 21%). ^{1}H NMR (400 MHz, $CDCl_3$) δ 8.94 (s, 1H), 8.12 – 7.85 (m, 2H), 7.56 – 7.28 (m, 6H), 6.92 (br s, 1H), 5.24 (s, 2H), 3.63 – 3.50 (m, 2H), 2.85 (q, *J* = 7.4, 2H),

1.30 (t, J = 7.4, 3H), 1.16 – 1.07 (t, 3H). ^{13}C NMR (101 MHz, $CDCl_3$) δ 166.85, 157.94, 136.28, 128.73, 128.24, 127.58, 123.66, 105.95, 70.43, 35.11, 31.04, 15.04, 14.64. HRMS (EI) m/z calcd for $C_{21}H_{22}N_2O_2S$ $[M]^+$ 366.1402, found 366.1383.

6-(benzyloxy)-N,N-diethyl-4-(ethylsulfanyl)quinoline-3-carboxamide 14. 14 was prepared according to the previous method using diethylamine (16 µL, 0.15 mmol). The crude product was purified by flash chromatography (gradient from 100% hexane to 100% EtOAc) to give **14** as a off-white powder (15.1 mg, 27%). ^{1}H NMR (400 MHz, $CDCl_3$) δ 8.60 (s, 1H), 8.06 (d, J = 9.1, 1H), 7.91 (d, J = 2.8, 1H), 7.58 – 7.30 (m, 6H), 5.27 (d, J = 3.7, 2H), 3.97 – 3.81 (m, 1H), 3.46 – 3.35 (m, 1H), 3.14 (q, J = 7.1, 2H), 3.00 – 2.78 (m, 2H), 1.33 (t, J = 7.1, 3H), 1.16 (t, J = 7.4, 3H), 1.06 (t, J = 7.1, 3H). ^{13}C NMR (101 MHz, $CDCl_3$) δ 128.75, 128.24, 127.54, 123.46, 105.40, 70.46, 43.01, 39.13, 30.76, 15.22, 14.14, 12.57. HRMS (EI) m/z calcd for $C_{23}H_{26}N_2O_2S$ $[M]^+$ 394.1715, found 394.1712.

6-(benzyloxy)-4-(ethylsulfanyl)-3-[(pyrrolidin-1-yl)carbonyl]quinoline 15. 15 was prepared according to the previous method using pyrrolidine (13 µL, 0.15 mmol). The crude product was purified by flash chromatography (gradient from 70/30 *n*-hexane/EtOAc to 100% EtOAc) to give **15** as a white powder (5.4 mg, 21%). ^{1}H NMR (400 MHz, $CDCl_3$) δ 8.63 (s, 1H), 8.09 (d, J = 9.2, 1H), 7.91 (d, J = 2.7, 1H), 7.67 – 7.31 (m, 6H), 5.27 (s, 2H), 3.73 (t, J = 7.0, 2H), 3.34 – 3.10 (m, 2H), 3.02 – 2.81 (m, 2H), 2.03 – 1.95 (m, 2H), 1.94 – 1.88 (m, 2H), 1.17 (t, J = 7.4, 3H). ^{13}C NMR (101 MHz, $CDCl_3$) δ 136.23, 128.75, 128.27, 127.57, 123.74, 105.37, 70.48, 48.19, 45.79, 30.55, 25.97, 24.53, 15.29. HRMS (ESI) m/z calcd for $C_{23}H_{24}N_2O_2S$ $[M + H]^+$ 393.1637, found 393.1655.

6-(benzyloxy)-4-(ethylthio)-*N*-(2-hydroxyethyl)quinoline-3-carboxamide 18. 18 was prepared according to the previous method using 2-ethanolamine (9 µL, 0.15 mmol). The crude product was purified by reversed-phase semi-preparative HPLC to give **18** as a white powder (4.5 mg, 7%). ^{1}H NMR (400 MHz, $CDCl_3$) δ 8.93 (s, 1H), 8.06 (d, J = 9.2, 1H), 7.91 (d, J = 2.6, 1H), 7.44 (m, 6H), 5.27 (s, 2H), 3.92 (t, J = 5.1, 2H), 3.71 (m, 2H), 2.28 (q, J = 7.4, 2H), 1.16 (t, J = 7.4, 3H). ^{13}C NMR (101 MHz, $CDCl_3$) δ 158.25, 146.31, 136.31, 128.90, 128.43, 127.71, 124.28, 105.98, 70.62, 62.08, 43.13, 31.28, 15.15. HRMS (ESI) m/z calcd for $C_{21}H_{22}N_2O_3S$ $[M + H]^+$ 383.1429, found 383.1445.

6-(benzyloxy)-4-(ethylthio)-*N*-phenylquinoline-3-carboxamide 16. To a solution of $AlMe_3$ (2 M in hexanes, 108 µL, 0.21 mmol) in dry toluene (1.5 mL), at -15 °C, was added aniline (19.1 µL, 0.21 mmol). The resulting solution was stirred for 20 min at -15 °C and 45 min at room temperature. **7** (70 mg, 0.19 mmol) was added and the mixture was stirred at 110 °C for 24 h. Water (2 mL) and sodium hydroxide 10% (m/v) (0.5 mL) were added to the solution and the product was extracted with ethyl acetate. The organic phase was washed with brine, dried and concentrated under reduced pressure. The residue was purified by flash chromatography (gradient from 100% *n*-hexane to 40/60 *n*-hexane/EtOAc) to give **16** as a pale yellow powder (12.4 mg, 14%). ^{1}H NMR (400 MHz, $CDCl_3$) δ 9.13 (s, 1H), 9.00 (s, 1H), 8.02 (d, 1H), 7.90 (d, 1H), 7.73 (d, 2H), 7.49 (m, 8H), 7.20 (m, 1H), 5.25 (s, 2H), 2.90 (q, 2H), 1.14 (t, 3H). ^{13}C NMR (101 MHz, $CDCl_3$) δ 136.05, 128.58, 128.10, 127.40, 123.56, 105.20, 70.31, 48.02, 45.61, 30.38, 25.80, 24.35, 15.11.

6-(benzyloxy)-4-(ethylthio)-*N*-(thiazol-2-yl)quinoline-3-carboxamide 17. 17 was prepared according to the previous method using 2-aminothiazole (20.3 mg, 0.21 mmol). The crude product was purified by flash chromatography (gradient from 70/30 *n*-hexane/EtOAc to 100% EtOAc) to give **17** as a yellow powder (16.9 mg, 21%). ^{1}H NMR (400 MHz, $CDCl_3$) δ 9.02 (s, 1H), 8.13 (d, 1H), 7.93 (d, 1H), 7.50 (m, 6H), 6.79 (d, 1H), 6.50 (d, 1H) 5.29 (s, 2H), 2.79 (q, 2H), 1.08 (t, 3H). ^{13}C NMR (101 MHz, $CDCl_3$) δ 164.70, 158.97, 158,29; 146.13; 144.75, 140.76, 136.65, 136.12, 131.99, 131.78, 130.38, 128.78, 127.54, 124.63, 113.88, 105.74, 70.51, 31.23, 15.30. MS (ESI) m/z calcd for $C_{22}H_{19}N_3O_2S$ $[M+H]^+$ 422.54, found 422.19.

(6-(benzyloxy)-4-(ethylthio)quinolin-3-yl)(morpholino)methanone 19. 19 was prepared according to the previous method using morpholine (18 µL, 0.21 mmol). The crude product was purified by flash chromatography (gradient from 60/40 *n*-hexane/EtOAc to 100% EtOAc) to give **17** as a yellow oil (3 mg, 3%). ^{1}H NMR (400 MHz, $CDCl_3$) δ 8.60 (s, 1H), 8.05 (d, *J* = 9.2, 1H), 7.88 (s, 1H), 7.50 (m, 6H), 5.29 (s, 2H), 3.85 (m, 4H), 3.60 (m, 2H), 3.22 (m, 2H), 2.88 (m, 2H), 1.14 (t, *J* = 7.4, 3H). ^{13}C NMR (101 MHz, $CDCl_3$) δ 166.79, 157.91, 144.43, 143.90, 138.36, 136.05, 134.60, 131.44, 130.51, 128.04, 127.30, 123.44, 105.12, 70.25, 66.56, 53.23, 47.09, 41.94, 30.59, 15.05. HRMS (ESI) *m*/*z* calcd for $C_{23}H_{24}N_2O_3S$ $[M+H]^+$ 409.1586, found 409.1573.

Ethyl 4-(ethylthio)-6-hydroxyquinoline-3-carboxylate 20. To a solution of **7** (2.41 g, 6.56 mmol) in TFA (20 mL) was added methanesulfonic acid (2 mL, 30.7 mmol). The mixture was stirred at 60 °C for 4 h. The TFA was removed under reduced pressure. The residue was carefully neutralized to pH = 7 with saturated $NaHCO_3$ and then, extracted with DCM. The organic layer was washed with water and brine, dried over $MgSO_4$ and evaporated. Purification by flash chromatography (gradient from 100% DCM to 95/5 DCM/MeOH) afforded **20** as a yellow powder (1.41 g, 77%). ^{1}H NMR (400 MHz, DMSO) δ 10.45 (s, 1H), 8.74 (s, 1H), 7.98 (d, *J* = 9.0,1H), 7.78 (d, *J* = 2.6, 1H), 7.44 (dd, *J* = 9.0, 2.6, 1H), 4.42 (q, *J* = 7.1, 2H), 2.96 (q, *J* = 7.4, 2H), 1.38 (t, *J* = 7.1, 3H), 1.10 (t, *J* = 7.4, 3H).

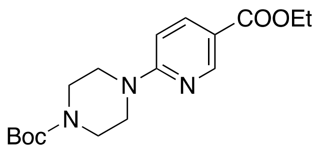

***tert*-butyl 4-(5-(ethoxycarbonyl)pyridin-2-yl)piperazine-1-carboxylate 21a.** Ethyl 6-chloronicotinate (371 mg, 2.0 mmol), 1-Boc-piperazine (372.5 mg, 2.0 mmol) and DIPEA (419 µL, 2.4 mmol) were dissolved in DMSO (4 mL) and stirred at 110 °C for 16 h. The mixture was diluted with EtOAc, washed with water and brine, dried over MgSO4 and concentrated under reduced pressure. The residue was purified by flash chromatography (gradient from 100% hexane to 70/30 hexane/EtOAc) to give a white solid (575 mg, 86%). ^{1}H NMR (400 MHz, $CDCl_3$) δ 8.80 (dd, *J* = 0.6, 2.3, 1H), 8.04 (dd, *J* = 2.3, 9.0,1H), 6.58 (d, *J* = 9;0, 1H), 4.33 (q, *J* = 7.1, 2H), 3.76 – 3.61 (m, 4H), 3.60 – 3.47 (m, 4H), 1.55 – 1.42 (s, 9H), 1.37 (t, *J* = 7.1, 3H).

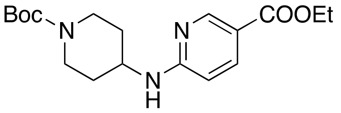

Ethyl 6-((1-(*tert*-butoxycarbonyl)piperidin-4-yl)amino)nicotinate 22a. 22a was prepared according to the above method from 6-chloronicotinate (371 mg, 2.0 mmol), 4-amino-1-Boc-piperidine (400.5 mg, 2.2 mmol) and DIPEA (419 µL, 2.4 mmol). Purification was performed with a gradient from 100% hexane to 60/40 hexane/EtOAC. White powder (235 mg, 34%). ^{1}H NMR (400 MHz, MeOD) δ 8.60 (dd, *J* = 0.6, 2.3, 1H), 7.88 (dd, *J* = 2.3, 8.9, 1H), 6.50 (dd, *J* = 0.6, 8.9, 1H), 4.30 (q, *J* = 7.1, 2H), 4.10 – 3.95 (m, 3H), 3.06 – 2.90 (m, 2H), 2.02 – 1.92 (m, 2H), 1.47 (s, 9H), 1.43 – 1.27 (m, 5H).

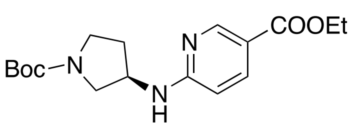

(S)-Ethyl 6-((1-(*tert*-butoxycarbonyl)pyrrolidin-3-yl)amino)nicotinate 23a. 23a was prepared according to method X from 6-chloronicotinate (371 mg, 2.0 mmol), (S)-1-*tert*-butoxycarbonyl-3-aminopyrrolidine (402 µL, 2.2 mmol) and DIPEA (419 µL, 2.4 mmol). Purification was performed with a gradient from 100% hexane to 60/40 hexane/EtOAC. Yellow oil (551 mg, 82%). ^{1}H NMR (400 MHz, $CDCl_3$) δ 8.75 (s, 1H), 8.01 (dd, *J* = 8.8, 2.2, 1H), 6.39 (d, *J* = 8.8, 1H), 5.19 (br d, 1H), 4.53 – 4.38 (m, 1H), 4.33 (q, *J* = 7.1, 2H), 3.73 (dd, *J* = 11.3, 6.0, 1H), 3.57 – 3.38 (m, 2H), 3.38 – 3.14 (m, 1H), 2.35 – 2.19 (m, 1H), 2.00 – 1.81 (m, 1H), 1.45 (s, 9H), 1.36 (t, *J* = 7.1, 3H).

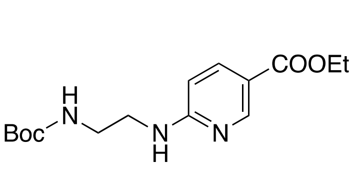

Ethyl 6-((2-((tert-butoxycarbonyl)amino)ethyl)amino)nicotinate 24a. **24a** was prepared according to the above method from 6-chloronicotinate (371 mg,

2.0 mmol), *N*-Boc-ethylenediamine (348.2 mg, 2.2 mmol) and DIPEA (419 µL, 2.4 mmol). Purification was performed with a gradient from 100% hexane to 50/50 hexane/EtOACto give a beige powder (297 mg, 48%). ^{1}H NMR (400 MHz, MeOD) δ 8.60 (d, *J* = 2.1, 1H), 7.90 (dd, *J* = 8.9, 2.1, 1H), 6.54 – 6.50 (d, *J* = 8.9, 1H), 4.31 (q, *J* = 7.1, 2H), 3.45 (t, *J* = 6.2, 2H), 3.25 (t, *J* = 6.2, 2H), 1.42 (s, 9H), 1.36 (d, *J* = 7.1, 3H).

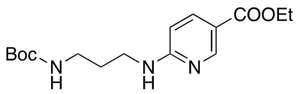

Ethyl 6-((3-((tert-butoxycarbonyl)amino)propyl)amino)nicotinate 25a. **25a** was prepared according to the above method from 6-chloronicotinate (371 mg, 2.0 mmol), *N*-Boc-1,3-propanediamine (384 µL, 2.2 mmol) and DIPEA (419 µL, 2.4 mmol). Purification was performed with a gradient from 100% hexane to 60/40 hexane/EtOAC. Yellow oil (498 mg, 77%). ^{1}H NMR (400 MHz, $CDCl_3$) δ 8.73 (d, *J* = 2.0, 1H), 7.96 (dd, *J* = 8.8, 2.0, 1H), 6.38 (d, *J* = 8.8, 1H), 5.55 (br s, 1H), 4.92 (br s, 1H), 4.32 (q, *J* = 7.1, 2H), 3.54 – 3.37 (m, 2H), 3.27 – 3.11 (m, 2H), 1.84 – 1.67 (m, 2H), 1.45 (s, 9H), 1.36 (t, *J* = 7.1, 3H). ^{13}C NMR (101 MHz, $CDCl_3$) δ 165.75, 160.39, 150.88, 138.14, 115.05, 106.55, 60.25, 60.22, 53.24, 38.27, 37.30, 29.88, 28.23, 20.88, 14.22, 14.03.

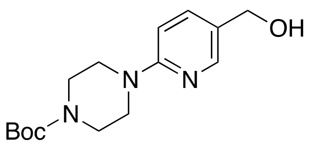

***tert*-butyl 4-(5-(hydroxymethyl)pyridin-2-yl)piperazine-1-carboxylate 21b. 21a** (200 mg, 0.596 mmol) was dissolved in THF (5 mL) and the solution was cooled to 0 °C. $LiAlH_4$ (113 mg, 2.98 mmol) was added and the reaction mixture was stirred for 30 min at 0 °C and for 1 h at room temperature. Ice and 10 mL of NaOH 10% (m/v) were added. The mixture was extracted with ethyl acetate, washed with brine, dried over MgSO4 and concentrated under reduced pressure. Purification over silica (gradient from 100% DCM to 95/10 DCM/MeOH) gave **21b** as a white powder (145 mg, 99%). ^{1}H NMR (400 MHz, $CDCl_3$) δ 8.15 (d, *J* = 2.3, 1H), 7.57 (dd, *J* = 8.7, 2.3, 1H), 6.67 (d, *J* = 8.7, 1H), 4.57 (s, 2H), 3.54 (br s, 8H), 1.48 (s, 9H). ^{13}C NMR (101 MHz, $CDCl_3$) δ 159.05, 154.84, 147.22, 137.61, 125.85, 107.15, 80.02, 62.63, 45.21, 28.43.

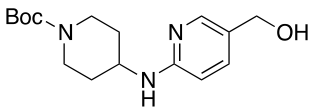

***tert*-butyl 4-((5-(hydroxymethyl)pyridin-2-yl)amino)piperidine-1-carboxylate 22b.** 22b was prepared according to the previous method from **22a** (235 mg, 0.67 mmol) and $LiAlH_4$ (76.6 mg, 2.02 mmol). Yellow solid (135 mg, 65%). ^{1}H NMR (400 MHz, MeOD) δ 7.89 (d, *J* = 2.0, 1H), 7.44 (dd, *J* = 8.6, 2.0, 1H), 6.53 (d, *J* = 8.6, 1H), 4.43 (s, 2H), 4.12 – 3.98 (m, 2H), 3.08 – 2.85 (m, 2H), 2.02 – 1.92 (m, 2H), 1.46 (s, 9H), 1.41 – 1.27 (m, 2H).

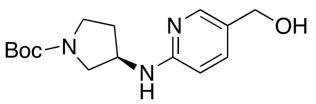

(S)-*tert*-butyl 3-((5-(hydroxymethyl)pyridin-2-yl)amino)pyrrolidine-1-carboxylate 23b. 23b was prepared according to the previous method from **23a** (351 mg, 1.05 mmol) and $LiAlH_4$ (119 mg, 3.14 mmol). Yellow solid (124 mg, 40%). ^{1}H NMR (400 MHz, MeOD) δ 7.93 (d, *J* = 2.1, 1H), 7.46 (dd, *J* = 8.6, 2.1, 1H), 6.56 (d, *J* = 8.6, 1H), 4.44 (s, 2H), 4.39 – 4.31 (m, 1H), 3.70 – 3.62 (m, 1H), 3.55 – 3.37 (m, 2H), 3.26 – 3.18 (m, 1H), 2.28 – 2.14 (m, 1H), 1.97 – 1.85 (m, 1H), 1.46 (s, 9H).

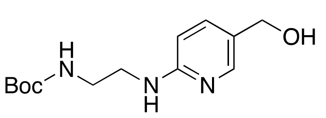

***tert*-butyl (2-((5-(hydroxymethyl)pyridin-2-yl)amino)ethyl)carbamate 24b**. **24b** was prepared according to the previous method from **24a** (247 mg, 0.92 mmol) and $LiAlH_4$ (105 mg, 2.77 mmol). White powder (201 mg, 82%). ^{1}H NMR (400 MHz, $CDCl_3$) δ 7.95 (d, *J* = 2.3, 1H), 7.45 (dd, *J* = 8.6, 2.3, 1H), 6.41 (d, *J* = 8.6, 1H), 5.16 (s, 1H), 5.08 (s, 1H), 4.50 (s, 2H), 3.46 – 3.38 (m, 2H), 3.38 – 3.26 (m, 2H), 1.42 (s, 9H). ^{13}C NMR (101 MHz, $CDCl_3$) δ 158.21, 146.72, 137.86, 125.29, 107.61, 62.70, 42.44, 40.47, 28.40.

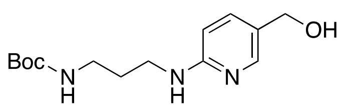

***tert*-butyl (3-((5-(hydroxymethyl)pyridin-2-yl)amino)propyl)carbamate 25b**. **25b** was prepared according to the previous method from **24a** (365 mg, 1.13 mmol) and $LiAlH_4$ (128 mg, 3.39 mmol). Colourless oil (320 mg, quant.). ^{1}H NMR (400 MHz, MeOD) δ 7.89 (d, J = 2.1, 1H), 7.45 (dd, J = 8.6, 2.1, 1H), 6.52 (d, J = 8.6, 1H), 4.43 (s, 2H), 3.30 (t, 2H + MeOH), 3.13 (t, J = 6.8, 2H), 1.74 (p, J = 6.8, 2H), 1.44 (s, 9H).

Ethyl 4-(ethylthio)-6-((6-(piperazin-1-yl)pyridin-3-yl)methoxy)quinoline-3-carboxylate 21. 20 (42 mg, 0.15 mmol), 6-(4-Boc-piperazin-1-yl)pyridin-3-yl)methanol (88 mg, 0.30 mmol) and triphenylphosphine (78.9 mg, 0.30 mmol) were dissolved in THF (2 mL) and the solution was cooled to 0 °C. To this solution was added DEAD (42.4 µL, 0.30 mmol). The reaction was stirred at room temperature overnight. The solvent was evaporated and the residue was purified by flash chromatography (gradient from 95/5 to 60/40 *n*-hexane/EtOAc). The Boc-protected intermediate was treated with TFA 20% (v/v) in DCM for 2 h at RT. Solvents were removed and the crude product was purified by semi-preparative HPLC/MS (50% to 98% MeOH in 10 min) to give **21** as a yellow oil (16 mg, 23% over 2 steps). ^{1}H NMR (400 MHz, $CDCl_3$) δ 9.97 (br s, 1H), 8.99 (s, 1H), 8.38 (d, 1H), 8.21 (d, 1H), 8.03 (d, 1H), 7.77 (dd, 1H), 7.57 (dd, 1H), 6.81 (d, 1H), 5.19 (s, 2H), 4.53 (q, 2H), 3.95 (s, 4H), 3.34 (s, 4H), 3.04 (q, 2H), 1.49 (t, 3H), 1.27 (t, 4H). ^{13}C NMR (101 MHz, $CDCl_3$) δ 165.93, 158.21, 147.34, 144.65, 138.90, 129.63, 125.22, 122.18, 107.70, 105.80, 67.91, 62.40, 42.95, 42.58, 31.22, 14.98, 14.25. HRMS (ESI) *m/z* calcd for $C_{24}H_{28}N_4O_3S$ $[M + H]^+$ 453.1960, found 453.1955.

Ethyl 4-(ethylthio)-6-((6-(piperidin-4-ylamino)pyridin-3-yl)methoxy)quinoline-3-carboxylate 22. 22 was prepared according to the previous method from **20** (55.4 mg, 0.20 mmol), *tert*-butyl 4-((5-(hydroxymethyl)pyridin-2-yl)amino)piperidine-1-carboxylate (123.0 mg, 0.40 mmol), triphenylphosphine (105.2 mg, 0.40 mmol) and DEAD (62.8 µL, 0.40 mmol) in THF (3 mL). After Boc-deprotection, and purification by semi-preparative HPLC/MS (50% to 98% MeOH in 10 min), **22** was isolated as a yellow powder (6.6 mg, 7% over 2 steps). ^{1}H NMR (400 MHz, MeOD) δ 8.76 (s, 1H), 8.35 (br s, 1H), 8.13 (d, J = 1.9, 1H), 8.05 – 7.99 (m, 2H), 7.61 (dd, J = 9.2, 2.5, 2H), 7.56 (dd, J = 9.2, 2.5, 1H), 6.61 (d, J = 8.6, 1H), 5.17 (s, 2H), 4.48 (q, J = 7.1, 2H), 4.07 – 3.94 (m, 1H), 3.44 (dt, J = 13.0, 3.4, 2H), 3.14 (td, J = 13.0, 3.4, 2H), 2.95 (q, J = 7.4, 2H), 2.24 (dd, J = 14.3, 3.4, 2H), 1.79 – 1.64 (m, 2H), 1.44 (t, J = 7.1, 3H), 1.16 (t, J = 7.4, 3H). ^{13}C NMR (101 MHz, MeOD) δ 137.80, 124.28, 105.76, 67.97, 61.86, 42.81, 30.41, 28.59, 13.13. HRMS (ESI) *m/z* calcd for $C_{25}H_{30}N_4O_3S$ $[M + H]^+$ 467.2117, found 467.2128.

(S)-ethyl 4-(ethylthio)-6-((6-(pyrrolidin-3-ylamino)pyridin-3-yl)methoxy)quinoline-3-carboxylate 23. 23 was prepared according to the previous method from **20** (55.4 mg, 0.20 mmol), (S)-*tert*-butyl 3-((5-(hydroxymethyl)pyridin-2-yl)amino)pyrrolidine-1-carboxylate (117.3 mg, 0.40 mmol) and triphenylphosphine (105.2 mg, 0.40 mmol) and DEAD (62.8 µL, 0.40 mmol) in THF (3 mL). The Boc-protected intermediate was isolated by flash chromatography (gradient from 50/50% hexane/EtOAc to 100% EtOAc). Purification of the final product by semi-preparative HPLC/MS (50% to 98% MeOH in 10 min) gave **23** as a yellow powder (10.5 mg, 11% over 2 steps). ^{1}H NMR (400 MHz, MeOD) δ 8.83 (s, 1H), 8.19 (d, J = 1.6, 1H), 8.12 (dd, J = 9.2, 1.6, 1H), 8.06 (m, 2H), 7.63 (dd, J = 9.2, 2.8, 1H), 7.12 (d, J = 9.2, 1H), 5.28 (s, 2H), 4.64 – 4.55 (m, 1H), 4.49 (q, J = 7.1, 2H), 3.67 (dd, J = 12.5, 6.4, 1H), 3.60 – 3.37 (m, 3H), 3.04 (q, J = 7.4, 2H), 2.48 (m, 1H), 2.26 – 2.14 (m, 1H), 1.45 (t, J = 7.1, 3H), 1.20 (t, J = 7.4, 3H). ^{13}C NMR (101 MHz, MeOD) δ 145.09, 130.00, 124.28, 105.81, 66.35, 62.01, 51.21, 49.61, 44.13, 30.55, 30.05, 13.89, 13.11. HRMS (ESI) *m/z* calcd for $C_{24}H_{28}N_4O_3S$ $[M + H]^+$ 453.1960, found 453.1964.

Ethyl 6-((6-((2-aminoethyl)amino)pyridin-3-yl)methoxy)-4-(ethylthio)quinoline-3-carboxylate 24. 24 was prepared according to the previous method from **20** (55.4 mg, 0.20 mmol), (S)-*tert*-butyl 3-((5-(hydroxymethyl)pyridin-2-yl)amino)pyrrolidine-1-carboxylate (105.2 mg, 0.40 mmol) and triphenylphosphine

(105.2 mg, 0.40 mmol) and DEAD (62.8 µL, 0.40 mmol) in THF (3 mL). **24** was isolated as a yellow powder (4.2 mg, 5% over 2 steps). ^{1}H NMR (400 MHz, MeOD) δ 8.87 (s, 1H), 8.21 (s, 1H), 8.15 (dd, *J* = 9.2, 2.5, 1H), 8.09 (t, *J* = 6.2, 2H), 7.66 (dd, *J* = 9.2, 2.5, 1H), 7.16 (d, *J* = 9.2, 1H), 5.31 (s, 2H), 4.52 (q, *J* = 7.1, 2H), 3.77 (t, *J* = 6.1, 2H), 3.29 (t, *J* = 6.1, 2H), 3.07 (q, *J* = 7.4, 2H), 1.48 (t, *J* = 7.1, 3H), 1.23 (t, *J* = 7.4, 3H). ^{13}C NMR (101 MHz, MeOD) 144.91, 136.72, 129.83, 124.19, 112.28, 105.60, 66.06, 61.70, 38.94, 37.75, 13.38, 12.71. HRMS (ESI) *m/z* calcd for $C_{22}H_{26}N_4O_3S$ $[M + H]^+$ 427.1804, found 427.1788.

Ethyl 6-((6-((2-aminoethyl)amino)pyridin-3-yl)methoxy)-4-(ethylthio)quinoline-3-carboxylate 24. **24** was prepared according to the previous method from (55.4 mg, 0.20 mmol), *tert*-butyl (2-((5-(hydroxymethyl)pyridin-2-yl)amino)ethyl)carbamate (106.8 mg, 0.40 mmol) and triphenylphosphine (105.2 mg, 0.40 mmol) and DEAD (62.8 µL, 0.40 mmol) in THF (3 mL). The Boc-protected intermediate was isolated by flash chromatography (gradient from 50/50% hexane/EtOAc to 100% EtOAc). Purification of the final product by semi-preparative HPLC/MS (50% to 98% MeOH in 10 min) gave **24** as a yellow powder (4.2 mg, 5% over 2 steps). ^{1}H NMR (400 MHz, MeOD) δ 8.87 (s, 1H), 8.21 (s, 1H), 8.15 (dd, *J* = 9.2, 2.5, 1H), 8.09 (t, *J* = 6.2, 2H), 7.66 (dd, *J* = 9.2, 2.5, 1H), 7.16 (d, *J* = 9.2, 1H), 5.31 (s, 2H), 4.52 (q, *J* = 7.1, 2H), 3.77 (t, *J* = 6.1, 2H), 3.29 (t, *J* = 6.1, 2H), 3.07 (q, *J* = 7.4, 2H), 1.48 (t, *J* = 7.1, 3H), 1.23 (t, *J* = 7.4, 3H). ^{13}C NMR (101 MHz, MeOD) 144.91, 136.72, 129.83, 124.19, 112.28, 105.60, 66.06, 61.70, 38.94, 37.75, 13.38, 12.71. HRMS (ESI) *m/z* calcd for $C_{22}H_{26}N_4O_3S$ $[M + H]^+$ 427.1804, found 427.1788.

Ethyl 6-((6-((3-aminopropyl)amino)pyridin-3-yl)methoxy)-4-(ethylthio)quinoline-3-carboxylate 25. **20** (55.4 mg, 0.20 mmol), tert-butyl (3-((5-(hydroxymethyl)pyridin-2-yl)amino)propyl)carbamate (112.5 mg, 0.40 mmol) and triphenylphosphine (105.2 mg, 0.40 mmol) were dissolved in THF (3 mL) and the solution was cooled to 0 °C. To this solution was added DEAD (62.8 µL, 0.40 mmol). The reaction was stirred at room temperature overnight. The solvent was evaporated and the residue was purified by flash chromatography (gradient from 100% DCM to 90/10 DCM/MeOH). The Boc-protected intermediate was treated with TFA 20% (v/v) in DCM for 40 min at RT. Solvents were removed and the crude product was purified by semi-preparative HPLC/MS (50% to 98% MeOH in 10 min) to give **25** as a yellow oil (6 mg, 7% over 2 steps). ^{1}H NMR (400 MHz, $CDCl_3$) δ 9.20 (br s, 1H), 8.84 (br s, 1H), 8.65 – 7.78 (m, 7H), 7.41 (d, *J* = 7.9, 1H), 7.00 (s, 1H), 5.03 (s, 2H), 4.44 (q, *J* = 7.1, 2H), 3.50 (s, 2H), 3.16 (s, 2H), 2.94 (q, *J* = 7.4, 2H), 2.12 (s, 2H), 1.41 (t, *J* = 7.1, 3H), 1.17 (t, *J* = 7.4, 3H). ^{13}C NMR (101 MHz, $CDCl_3$) 146.06, 143.02, 134.59, 131.35, 123.52, 105.48, 65.86, 61.82, 39.21, 36.87, 30.68, 25.29, 14.49, 13.75 HRMS (ESI) *m/z* calcd for $C_{23}H_{28}N_4O_3S$ $[M + H]^+$ 441.1960, found 441.1945.

Ethyl 4-((2-cyanoethyl)thio)-6-((6-(piperazin-1-yl)pyridin-3-yl)methoxy)quinoline-3-carboxylate 26. To a solution of **4** (2.0 g, 5.09 mmol) in DCM (20 mL) at 0 °C was added a solution of boron tribromide in DCM (1.0M, 6.1 mL, 6.11 mmol). The mixture was stirred at 0 °C for 30min and was allowed to warm to RT. The solvents were evaporated and the crude product dissolved in methanol then absorbed onto silica. Purification by flash chromatography (gradient from 100% DCM to 90/10 DCM/MeOH) afforded ethyl 4-(2-cyanoethyl)thio)-6-hydroxyquinoline-3-carboxylate as a yellow powder (720 mg, 47%).

This intermediate (40.0 mg, 0.13 mmol), 6-(4-Boc-piperazin-1-yl)pyridin-3-yl)methanol (77.4 mg, 0.26 mmol) and triphenylphosphine (69.4 mg, 0.26 mmol) were dissolved in THF/DMF (2.5 mL) and the solution was cooled to 0 °C. To this solution was added DEAD (41.4 µL, 0.26 mmol). The reaction was stirred at room temperature overnight. The reaction mixture was diluted with ethyl acetate (20 mL) and washed with brine (3x 10mL). The organic layer was dried over $MgSO_4$ and evaporated to give a brown oil. The crude product was purified by flash chromatography (gradient from 1:1 to 4:1 ethyl acetate/hexane The Boc-protected intermediate was treated with TFA 20% (v/v) in DCM for 2h at RT. Solvents were removed and the crude product was purified by semi-preparative HPLC/MS (15% to 98% MeOH in 10 min) to give **26** as a yellow powder (21.2 mg, 37% over 2 steps). ^{1}H NMR (400 MHz, $CDCl_3$) δ

9.20 (br s, 1H), 8.84 (br s, 1H), 8.65 – 7.78 (m, 7H), 7.41 (d, *J* = 7.9, 1H), 7.00 (s, 1H), 5.03 (s, 2H), 4.44 (q, *J* = 7.1, 2H), 3.50 (s, 2H), 3.16 (s, 2H), 2.94 (q, *J* = 7.4, 2H), 2.12 (s, 2H), 1.41 (t, *J* = 7.1, 3H), 1.17 (t, *J* = 7.4, 3H). ^{13}C NMR (101 MHz, $CDCl_3$) 146.06, 143.02, 134.59, 131.35, 123.52, 105.48, 65.86, 61.82, 39.21, 36.87, 30.68, 25.29, 14.49, 13.75 HRMS (ESI) *m*/*z* calcd for $C_{23}H_{28}N_4O_3S$ $[M + H]^+$ 441.1960, found 441.1945.

ENZYME INHIBITION ASSAY

Final compounds were tested using a previously described fluorogenic 96-well plate assay for PvNMT,[2] PfNMT[3] and HsNMT1/2[4] activity. Data were elaborated using Microsoft Office Excel 2010 and IC_{50} values were determined by nonlinear regression fitting using GraFit 7.0 (Erithacus Software Ltd., U.K.). Apparent inhibition constants, K_i^{app}, were calculated from experimental IC_{50} using Cheng-Prussof equation.[5]

SUPPLEMENTARY FIGURE S1

Fig. S1 Chemical structure of different lead compounds targeting *N*-myristoyltransferases from *Plasmodium falciparum*,[6, 7] *Plasmodium vivax*,[6] Trypanosoma brucei[8] and *Leishmania donovani*.[9]

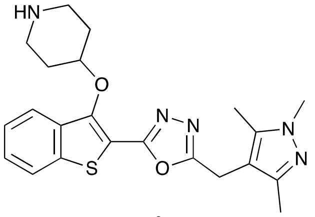

34c [6]
K_i PfNMT : 0.008 μM
K_i PvNMT : 0.002 μM

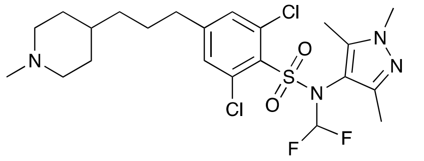

DDD100097 [8]
IC_{50} TbNMT : 2 nM

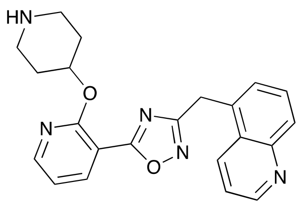

30 [7]
K_i PfNMT : 0.0017 μM

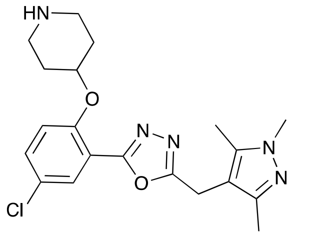

13 [9]
K_i LdNMT : 0.01 μM

SUPPLEMENTARY TABLE S1

Table S1. X-ray data collection and refinement statistics

**PDB accession code**	PvNMT-NHM-**1** **5g1z**	LmNMT-MyrCoA-**19** **5g20**	LmNMT-MyrCoA-**26** **5g21**	PvNMT-NHM-**26** **5g22**
Cell dimensions	57.40, 118.90, 175.30	47.72, 90.65, 52.91	48.68, 91.07, 53.57	57.97, 123.36, 179.37
Cell angles α, β,	90.0, 90.0, 90.0	90.0, 112.7, 90.0	90.0, 114.5, 90.0	90.0, 90.0, 90.0
Space Group	$P2_12_12_1$	$P2_1$	$P2_1$	$P2_12_12_1$
**Data collection**				
Beamline /	DLS i03 / 0.9763	DLS i04 / 0.9795	DLS i03 / 0.9763	DLS i03 / 0.9763
Detector type	Pilatus CMOS	ADSC CCD	Pilatus CMOS	Pilatus CMOS
Images x	1800 x 0.2	360 x 0.5	1100 x 0.2	1098 x 0.2
Resolution (Å)	59–1.50 (1.53–1.50) [a]	49–1.52 (1.55–1.52)	49–1.50 (1.53–1.50)	42–2.32 (2.45–2.32)
R_{sym} (%) [b]	7.2 (63.2)	5.8 (76.5)	4.2 (55.7)	19.4 (33.2)
$I/\sigma I$	13.3 (2.2)	10.0 (1.5)	13.8 (2.3)	7.1 (3.0)
Completeness	99.4 (99.3)	98.8 (97.5)	99.7 (99.1)	99.2 (97.6)
Redundancy	6.3 (4.3)	3.8 (3.8)	4.2 (3.9)	6.7 (4.2)
**Refinement**				
No. unique	191010	63023	67660	56204
R_{work} / R_{free} [c]	14.9 / 18.7	17.4 / 22.2	17.4 / 20.9	22.0 / 28.3
No. atoms	11889	3982	4073	10381
Protein	9963	3504	3565	9637
Ligand	66	29	34	102
Co-factor	192	63	63	192
Water	1632	381	376	439
B-factors (Å^2)				
All atoms	19.8	24.5	26.5	21.3
Protein	17.9	23.6	25.7	21.1
Ligand	23.9	25.6	21.2	30.2
Co-factor	13.9	16.3	17.3	15.7
Water	31.6	33.5	34.4	25.3
R.m.s.deviations				
Bond lengths	0.025	0.024	0.027	0.014
Bond angles	2.365	2.336	2.621	1.691

[a] Highest resolution shell is shown in parentheses.
[b] $R_{sym} = \Sigma_{\mathbf{h}}\Sigma_l\, |I_{\mathbf{h}l} - \langle I_{\mathbf{h}}\rangle| / \Sigma_{\mathbf{h}}\Sigma_l \langle I_{\mathbf{h}}\rangle$, where I_l is the lth observation of reflection h and $\langle I_{\mathbf{h}}\rangle$ is the weighted average intensity for all observations l of reflection h.
[c] $R_{cryst} = \sum||F_o| - |F_c||/\sum|F_o|$ where F_o and F_c are the observed and calculated structure factor amplitudes, respectively. R_{free} is the R_{cryst} calculated with 5% of the reflections omitted from refinement.
[d] Root-mean-square deviation of bond lengths or bond angles from ideal geometry.

SUPPLEMENTARY FIGURE S2

Fig. S2 Stereo figures: a) PvNMT-NHM-**1**, b) LmNMT-MyrCoA-**19**, c) PvNMT-NHM-**26**, d) LmNMT-MyrCoA-**26**.

a)

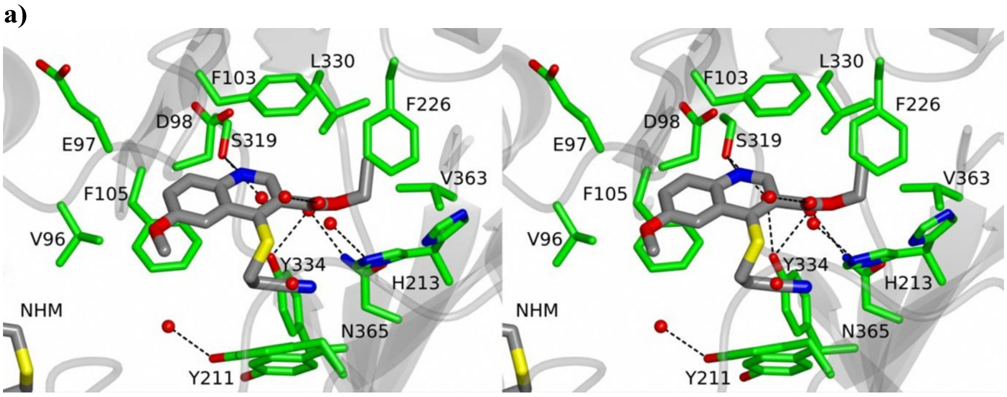

b)

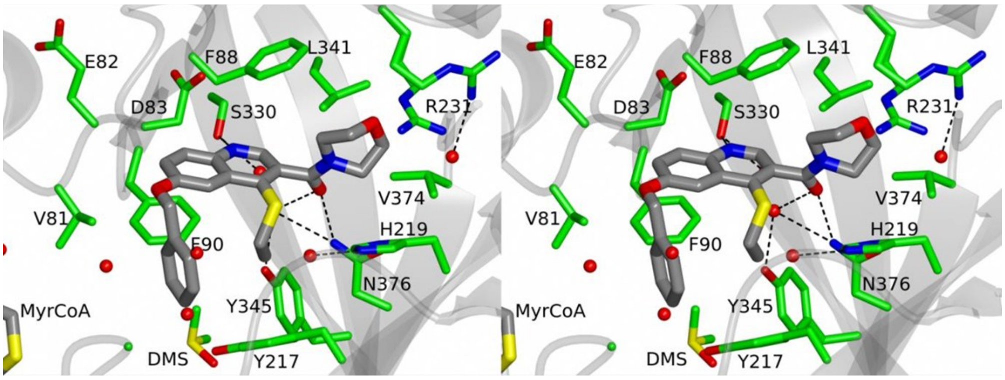

c)

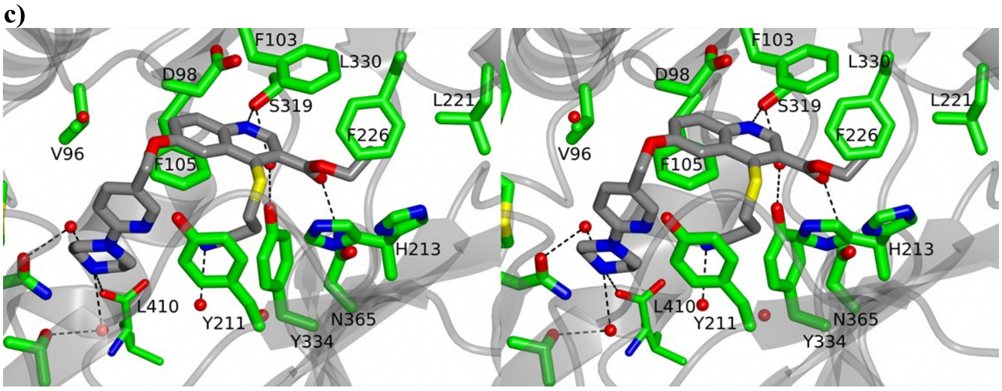

d)

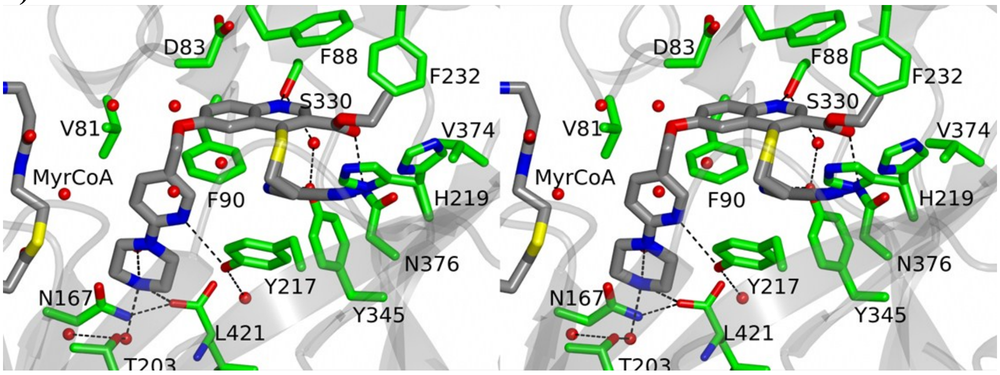

SUPPLEMENTARY FIGURE S3

Fig. S3 Superposition of PvNMT-NHM-**1** with LmNMT-MyrCoA-**19**: top) Stereo view of superposed protein chains, bottom) Stereo view of ligands. Superimposition of **1** (green carbons) in PvNMT with **19** (gray carbons) in LmNMT. This figure highlights the differences in orientation of the carbonyl groups between the amide (**19**) and ester (**1**) chains.

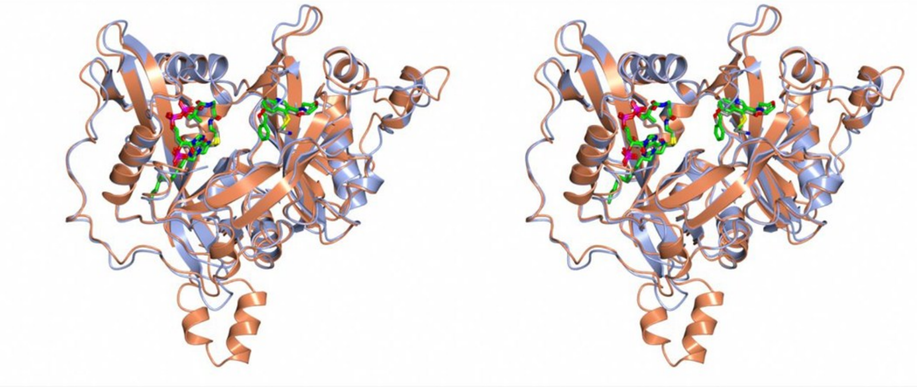

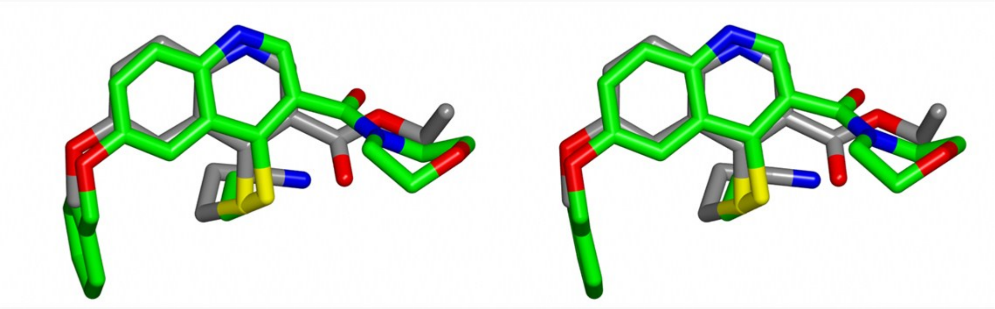

SUPPLEMENTARY FIGURE S4

Fig. S4 PvNMT-NHM-**26** a) superposition of ligand bound to three independent chains in the asymmetric unit of the crystal cell b) Pv (Chain B) superimposed onto Lm-Myr-**26** with an enlarged view of the ligands on the right. c) stereo view of the inhibitors together with Tyr211.

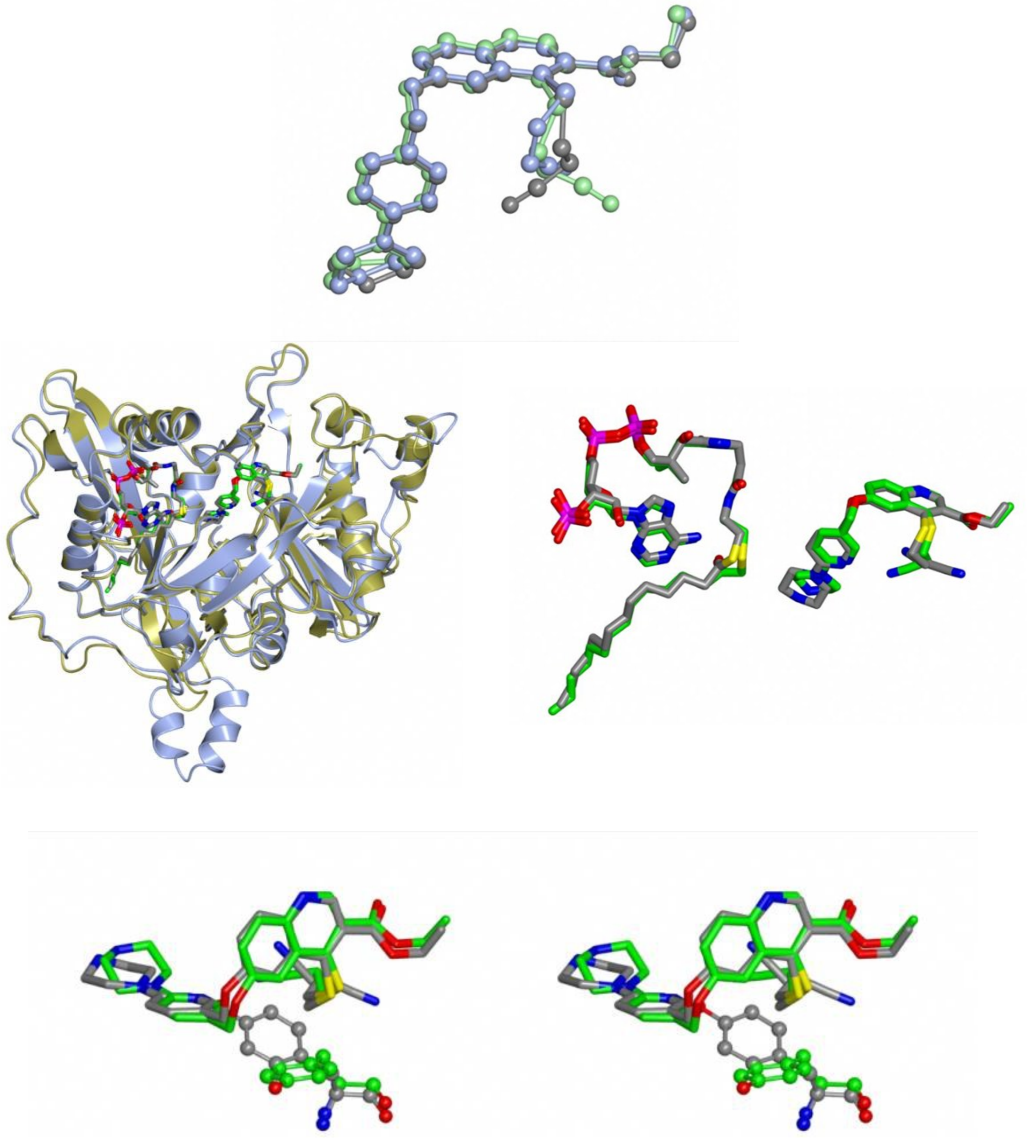

SUPPLEMENTARY FIGURE S5

Fig. S5 Orthogonal views of superposed ligands in complex with LmNMT. a) Compound **26** (green carbons) with DDU85646 (grey carbons, PDB 2WSA); b) Compound **19** (light-green carbons) with DDU85646; c) Compound **19** with Compound **26**. For stereo figures, see Figure S6.

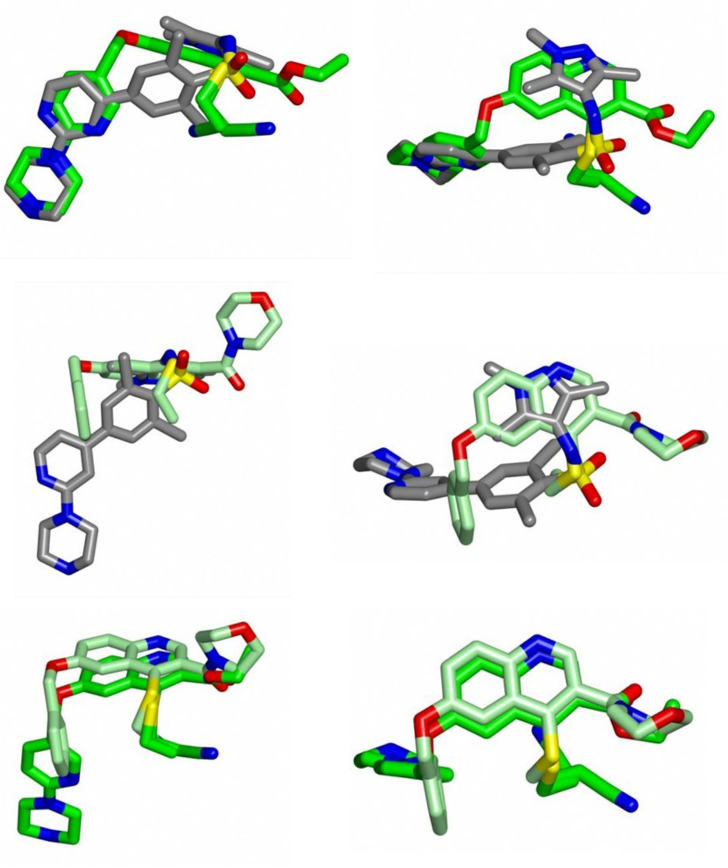

SUPPLEMENTARY FIGURE S6

Fig. S6 Stereo views of superposed ligands in complex with LmNMT. a) Compound **26** (green carbons) with DDU85646 (grey carbons, PDB 2WSA); b) Compound **19** (light-green carbons) with DDU85646; c) Compound **19** with Compound **26**.

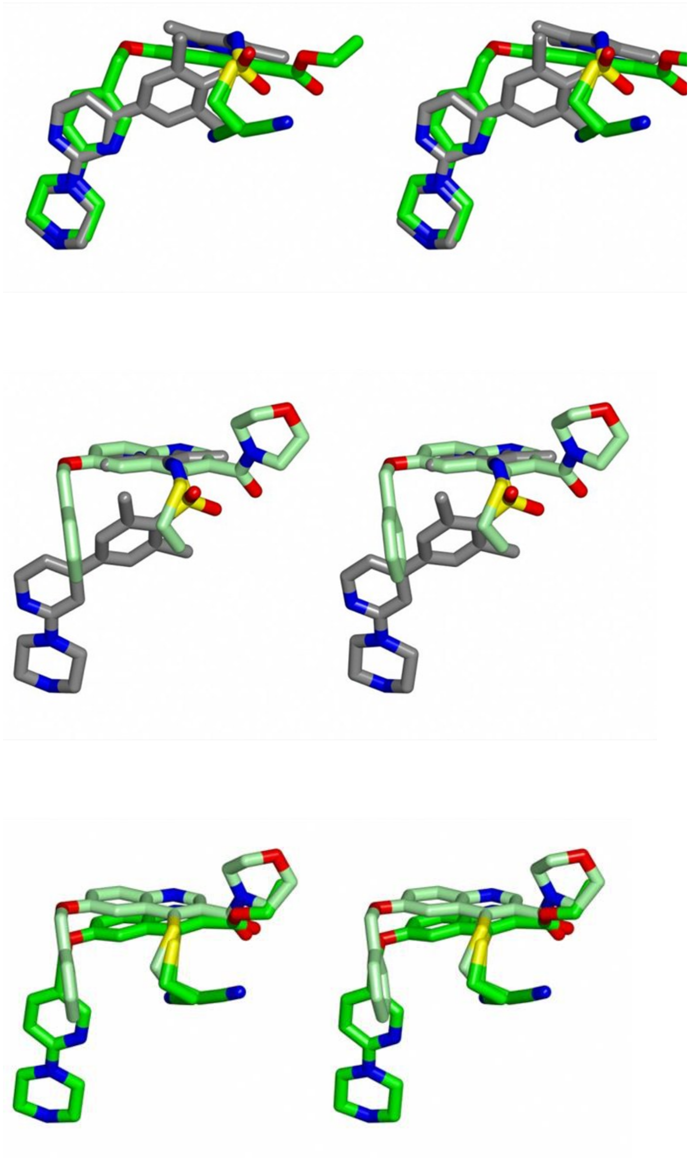

REFERENCES

1. J. Klose, C. B. Reese and Q. Song, *Tetrahedron*, 1997, **53**, 14411-14416.
2. V. Goncalves, J. A. Brannigan, D. Whalley, K. H. Ansell, B. Saxty, A. A. Holder, A. J. Wilkinson, E. W. Tate and R. J. Leatherbarrow, *J. Med. Chem.*, 2012, **55**, 3578-3582.
3. M. D. Rackham, J. A. Brannigan, D. K. Moss, Z. Yu, A. J. Wilkinson, A. A. Holder, E. W. Tate and R. J. Leatherbarrow, *J. Med. Chem.*, 2013, **56**, 371-375.
4. V. Goncalves, J. A. Brannigan, E. Thinon, T. O. Olaleye, R. Serwa, S. Lanzarone, A. J. Wilkinson, E. W. Tate and R. J. Leatherbarrow, *Anal. Biochem.*, 2012, **421**, 342-344.
5. Y.-C. Cheng and W. H. Prusoff, *Biochem. Pharmacol.*, 1973, **22**, 3099-3108.
6. M. D. Rackham, J. A. Brannigan, K. Rangachari, S. Meister, A. J. Wilkinson, A. A. Holder, R. J. Leatherbarrow and E. W. Tate, *J. Med. Chem.*, 2014, **57**, 2773-2788.
7. Z. Y. Yu, J. A. Brannigan, K. Rangachari, W. P. Heal, A. J. Wilkinson, A. A. Holder, R. J. Leatherbarrow and E. W. Tate, *Medchemcomm*, 2015, **6**, 1767-1772.
8. S. Brand, N. R. Norcross, S. Thompson, J. R. Harrison, V. C. Smith, D. A. Robinson, L. S. Torrie, S. P. McElroy, I. Hallyburton, S. Norval, P. Scullion, L. Stojanovski, F. R. C. Simeons, D. van Aalten, J. A. Frearson, R. Brenk, A. H. Fairlamb, M. A. J. Ferguson, P. G. Wyatt, I. H. Gilbert and K. D. Read, *J. Med. Chem.*, 2014, **57**, 9855-9869.
9. M. D. Rackham, Z. Y. Yu, J. A. Brannigan, W. P. Heal, D. Paape, K. V. Barker, A. J. Wilkinson, D. F. Smith, R. J. Leatherbarrow and E. W. Tate, *Medchemcomm*, 2015, **6**, 1761-1766.
